# Cathepsin S regulates antitumor immunity through autophagic degradation of PD-L1 in colorectal cancer cells

**DOI:** 10.1007/s00262-025-04140-x

**Published:** 2025-08-12

**Authors:** Sina Taheri Baghmisheh, Chung-Hsing Chen, Yu-Min Yeh, Peng-Chan Lin, Po-Chuan Chen, Ren-Hao Chan, Jui-Wen Kang, Chung-Ta Lee, Hui-Ju Tsai, Yu-Chen Fang, Chun Hei Antonio Cheung, Kwang-Yu Chang, Jang-Yang Chang, Shang-Hung Chen

**Affiliations:** 1 National Institute of Cancer Research, National Health Research Institutes, Tainan, Taiwan; 2 Department of Mathematics, University of Taipei, Taipei, Taiwan; 3 Department of Oncology, National Cheng Kung University Hospital, College of Medicine, National Cheng Kung University, Tainan, Taiwan; 4 Department of Genomic Medicine, College of Medicine, National Cheng Kung University Hospital, National Cheng Kung University, Tainan, Taiwan; 5 Division of Colorectal Surgery, Department of Surgery, National Cheng Kung University Hospital, College of Medicine, National Cheng Kung University, Tainan, Taiwan; 6 Department of Internal Medicine, National Cheng Kung University Hospital, College of Medicine, National Cheng Kung University, Tainan, Taiwan; 7 Department of Pathology, College of Medicine, National Cheng Kung University Hospital, National Cheng Kung University, Tainan, Taiwan; 8 Department of Pharmacology, College of Medicine, National Cheng Kung University, Tainan, Taiwan; 9 Institute of Basic Medical Sciences, College of Medicine, National Cheng Kung University, Tainan, Taiwan; 10 TMU Research Center of Cancer Translational Medicine, Taipei Cancer Center, Taipei Medical University Hospital, College of Medicine, Taipei Medical University, Taipei, Taiwan; 11 Institute of Biotechnology and Pharmaceutical Research, National Health Research Institutes, Miaoli, Taiwan

**Keywords:** CTSS, Colorectal cancer, Immune microenvironment, PD-L1, Autophagy

## Abstract

Colorectal cancer (CRC) is a major contributor to cancer-related mortality worldwide, highlighting the need to overcome its immunosuppressive tumor microenvironment. Cathepsin S (CTSS), a cysteine protease essential for MHC class II antigen presentation, has an unclear role in CRC immunity. This study investigated the impact of CTSS on PD-L1 expression and T-cell function in CRC. CTSS expression was analyzed in CRC tumor tissues and CTSS-deficient cell lines using immunohistochemistry, Western blotting, and flow cytometry. T-cell responses were assessed through granzyme B and IL-2 secretion assays, migration analysis, and gene set variation analysis (GSVA) of public datasets. Autophagy activity was evaluated via immunofluorescence, Western blotting, and lysosome isolation assays. An orthotopic CRC mouse model was used to study CTSS function in vivo. Key findings revealed that elevated CTSS expression correlated with higher PD-L1 levels in CRC tissues. CTSS suppression in CRC cells reduced PD-L1 expression while enhancing T-cell cytotoxicity and migration. GSVA further revealed an inverse correlation between CTSS expression and cytotoxic T-cell activity, alongside a strong association with autophagy-related pathways. Mechanistically, CTSS suppression in CRC cells promoted PD-L1 degradation by enhancing autophagic flux. In vivo, CTSS suppression inhibited tumor growth and enhanced CD8⁺ T-cell infiltration and activity. Anti-CD8 antibody treatment promoted tumor growth more significantly in CTSS-proficient CRC cells compared to CTSS-deficient cells. These findings demonstrate that CTSS regulates PD-L1 expression and T-cell cytotoxicity via autophagy-mediated pathways in CRC cells. Given ongoing development of CTSS inhibitors and autophagy modulators, targeting CTSS may offer a promising strategy to improve CRC immunotherapy.

**Supplementary Information:**

The online version contains supplementary material available at 10.1007/s00262-025-04140-x.

## Background

In 2020, more than 1.9 million new cases of colorectal cancer (CRC) were diagnosed and CRC accounted for over 900,000 deaths; CRC was thus the third most common cancer and the second leading cause of cancer-related mortality worldwide [1–3]. In Taiwan, changes in dietary and lifestyle habits over the past decade have contributed to a steady increase in CRC incidence [4]. According to the Health Promotion Administration (Ministry of Health and Welfare of Taiwan), approximately 16,000 new CRC cases and 6,000 CRC-related deaths were reported in 2021 [2]. Thus, CRC management is a critical public health priority both globally and in Taiwan. Recent advances in immunotherapy, particularly immune checkpoint inhibitors (ICIs) targeting programmed cell death 1 (PD-1) and its ligand PD-L1, have considerably influenced cancer treatment [5, 6]. Although the efficacy of these ICIs has been demonstrated in several malignancies, their clinical efficacy in CRC is largely limited to patients with tumors with high microsatellite instability (MSI-H) [7, 8]. Expanding the effectiveness of immunotherapy to a broader CRC population requires the identification of critical molecular targets involved in immune evasion.

In the tumor microenvironment (TME), tumor cells evade immune surveillance to sustain their growth [9]. CD8^+^ T-cells are essential for immune-mediated tumor elimination, but cancer cells can inhibit these cells’ cytotoxic function by activating immune checkpoints, including PD-1, cytotoxic T-lymphocyte-associated protein 4 (CTLA4), T-cell immunoglobulin and mucin-domain containing-3 (TIM-3), and lymphocyte-activation gene 3 (LAG-3) [10, 11]. Although immune checkpoints normally regulate immune responses to prevent overactivation, cancer cells exploit this mechanism to suppress CD8^+^ T-cell activity, leading to T-cell exhaustion [12]. The PD-1/PD-L1 axis is a key target in cancer immunotherapy [5, 6], with increasing evidence suggesting that PD-L1 expression on tumor cells can serve as a critical biomarker for predicting the efficacy of anti-PD-1/PD-L1 therapies [13, 14].

The lysosomal system plays a critical role in intracellular protein degradation through the action of various hydrolases [15]. Of the approximately 50 known lysosomal hydrolases, the aspartic, serine, and cysteine proteases—collectively known as “cathepsins”—are essential for maintaining intracellular homeostasis [16]. Cathepsin S (CTSS) has been implicated in immune modulation; it regulates the processing and presentation of major histocompatibility complex (MHC) class II antigens in antigen-presenting cells [17]. Although CTSS is upregulated in specific solid tumors, including CRC, its precise role in promoting tumor growth remains unclear [18, 19]. Emerging evidence suggests that CTSS may contribute to tumor progression by modulating immune responses within the TME [20–22]. These findings underscore the need for further investigation into the role of CTSS in CRC, particularly its potential influence on PD-L1 expression in tumor cells.

This study demonstrated that CTSS overexpression is associated with increased PD-L1 expression in CRC cells, as evidenced by in silico, in vitro, and in vivo analyses. Functional studies have further revealed that CTSS modulates cytotoxic T-lymphocyte activity, influencing CRC cell growth. Mechanistic investigations have indicated that CTSS suppression enhances autophagic flux in CRC cells, whereas autophagy inhibition restores PD-L1 expression in CTSS-deficient cells. To the best of our knowledge, this is the first study to identify CTSS as a regulator of CRC tumor immunity through autophagy modulation. The findings offer novel insights into CRC immune regulation and may inform the development of novel immunotherapy strategies.

## Materials and methods

### Reagents and antibodies

Anti-CTSS (#sc-74429) and ATG-7 (#sc-376212) antibodies were obtained from Santa Cruz Biotechnology (CA, USA). PD-L1 antibody (#13,684) was acquired from Cell Signaling Technology (MA, USA). LC3B (GeneTex, #GTX127375) was purchased from GeneTex (CA, USA), whereas SQSTM1 (P62; #ab91526) was obtained from Abcam (Cambridge, UK). Additionally, actin (#MAB1501), chloroquine (CQ; #C6628), and phytohemagglutinin (PHA; #L8754) were all purchased from Sigma-Aldrich (St. Louis, MO, USA).

### Cell culture

Human CRC cell lines SW480 and HT29, along with immortalized human T-cells (Jurkat cells), were used in this study. HT29 is KRAS wild-type and microsatellite stable (MSS), whereas SW480 is KRAS-mutant and also MSS [23]. These characteristics represent clinically relevant CRC subtypes with differing responses to targeted therapies such as anti-EGFR agents. The SW480 and Jurkat cells were obtained from the Bioresource Collection and Research Center (Hsinchu City, Taiwan), whereas the HT29 cells were purchased from the American Type Culture Collection (Rockville, MD, USA). Upon receipt of the cells, they were cultured and frozen as seed stocks, with passaging limited to 3 months before fresh stocks were thawed. The SW480 cells were cultured in Dulbecco’s modified Eagle medium, and the HT29 cells were cultured in RPMI 1640 medium, both at 37 °C with 5% CO_2_. The culture media were supplemented with 10% FBS, 100 μg/mL streptomycin sulfate, and 100 U/mL penicillin-G sodium. The cells were passaged every 2–3 days by using 0.05% trypsin and were subcultured at an initial density of 1 × 10^5^ cells/mL per 10-cm tissue dish.

### Bioinformatic analysis of CTSS expression

CTSS expression levels were analyzed using data from The Cancer Genome Atlas (TCGA) through the Human Protein Atlas and Gene Expression Profiling Interactive Analysis (GEPIA). GEPIA was used to compare CTSS expression in CRC versus normal colon mucosa tissues. Additionally, associations between CTSS expression and immune cell abundance were assessed using the TIMER2.0 database, which estimates abundance through statistical methods.

### Patients and tumor specimens

The use of CRC tissues in this study was approved by the Institutional Review Board of National Cheng Kung University Hospital (approval number: A-ER-109-547). A total of 92 patients with MSS CRC who underwent curative surgery followed by adjuvant oxaliplatin-based chemotherapy were included. The median follow-up was 1,750 days, and the tumor recurrence rate among the patients was approximately 22%.

### Immunohistochemical staining

Immunohistochemical (IHC) staining was performed on 4-µm-thick, formalin-fixed, paraffin-embedded tissue sections by using the Bond-Max Automated IHC Stainer (Leica Biosystems, Newcastle Ltd., Australia). Tissue sections were deparaffinized with xylene, pretreated with Epitope Retrieval Solution 2 (EDTA buffer, pH 9.0) at 100 °C for 30 min, and incubated at room temperature for 30 min with anti-CTSS antibodies (1:300) serving as primary antibodies. Following this incubation, the tissues were treated with the postprimary reagent and polymer by using the Bond Polymer Refine Detection Kit (Leica Biosystems, Newcastle Ltd., UK). The sections were then developed using 3,3′-diaminobenzidine chromogen for 10 min and counterstained with hematoxylin. CTSS expression levels were quantified using an H-score, calculated as H-score = ΣPi (i + 1), where “i” represents the staining intensity (0 to 3 +), and “Pi” denotes the percentage of tumor cells with various intensities (0% to 100%). PD-L1 staining was conducted through incubation with the primary antibody monoclonal mouse antihuman PD-L1 (Clone 22C3, Dako, 1:50) by using the Bond-Max Automated IHC Stainer in accordance with the manufacturer’s instructions. PD-L1 expression was assessed using the tumor proportion score (TPS), as outlined in the PD-L1 IHC 22C3 pharmDx package insert.

### Western blot analysis

Western blot analysis was conducted as previously described [24]. Briefly, cells were lysed in lysis buffer (Merck Millipore, Darmstadt, Germany), and proteins were extracted and separated through sodium dodecyl sulfate–polyacrylamide gel electrophoresis. The proteins were then transferred onto polyvinylidene fluoride membranes (Merck Millipore, Darmstadt, Germany). Immunoreactive bands were detected using the Western Lightning Plus-ECL Enhanced Chemiluminescence Substrate (PerkinElmer, Inc., Waltham, MA, USA) and visualized on Kodak X-Omat film (Kodak, Chalon/Paris, France). Band intensities were quantified using ImageJ software and normalized to actin levels to ensure consistency.

### Short hairpin RNA transfection

Lentiviral transduction was performed using the pLKO.1 lentiviral construct encoding short hairpin RNA (shRNA). CTSS shRNA plasmids (TRCN0000003695 and TRCN0000279838) and a control plasmid were obtained from the RNAi Core Academia Sinica (Taipei City, Taiwan). Lentiviruses were generated in 293 T cells through effectene (Qiagen)-mediated cotransfection of the pLKO.1 plasmid. Sixteen hours after transfection, the 293 T cells were rinsed with phosphate-buffered saline (PBS) and cultured in fresh growth media. Forty-eight hours after transfection, the lentiviral supernatant was harvested, centrifuged at 2000 × g for 5 min at 4 °C, and filtered through a 0.45-μM syringe filter. The SW480 and HT29 cells were then incubated with the lentiviral supernatant containing 5 μg/mL polybrene. Six hours after infection, the transduced cells were rinsed with PBS and cultured in fresh growth media. The lentiviral infection process was repeated the following day. The transduced cells were selected using 2 μg/mL puromycin for 48–72 h.

### Small interfering RNA transfection

Cancer cells were transfected with target-validated small interfering RNA (siRNA) oligos by using Lipofectamine 3000 (Thermo Fisher Scientific, L3000015) in accordance with the manufacturer’s protocol. Briefly, CRC cells were seeded in 6-cm dishes and cultured overnight in antibiotic-free medium. Scramble siRNA (Dharmacon, D-001206-13-05), ATG7-specific siRNA oligos (Santa Cruz, # sc-41447), or CTSS-specific siRNA (Ambion, #4,392,420) were diluted in Opti-MEM I medium (Thermo Fisher Scientific, # 11,058,021) without serum or in Opti-MEM I medium containing Lipofectamine 3000. After 20-min incubation at room temperature, the transfection mixture was added to the cells, and incubation was conducted for the designated period.

### CD8^+^ T-cell isolation, culture, and analysis

Human peripheral blood mononuclear cells (PBMCs) from healthy donors were used as effector cells following CD8^+^ T-cell isolation (Thermo Fisher Scientific, cat # 11348D), in accordance with the manufacturer’s instructions. The CD8^+^ T-cells were cultured in RPMI 1640 supplemented with 10% FBS, 1% penicillin–streptomycin–glutamine, 1% MEM nonessential amino acids solution, 1% HEPES, 1% sodium pyruvate, and 0.05 mM β-mercaptoethanol. T-cells were seeded at a density of 0.5 × 10⁶ cells per well in 24-well plates and activated using CD3/CD28 (Thermo Fisher Scientific, cat # 11131D) and interleukin-2 (IL-2, 30 U/mL). Supernatants were collected through centrifugation and filtered through a 0.22-μm steriflip (Millipore), after which IL-2 concentrations were quantified using a human IL-2 enzyme-linked immunosorbent assay (ELISA) kit (Invitrogen, cat # BMS221INST) in accordance with the manufacturer’s protocol.

### Growth inhibition assay

Cell viability was assessed using methylene blue staining, as previously described [24]. Briefly, cells in the logarithmic growth phase were seeded at a density of 1 × 10^4^ to 3 × 10^4^ cells per well in 24-well plates. After these cells had been exposed to cytotoxic T-cells, the impact of the T-cells on cancer cell growth was assessed using the methylene blue dye assay.

### Flow cytometry analysis

CRC cells were surface-stained with anti-CD8α FITC (BD Pharmingen, clone RPA-T8), after which they were fixed and permeabilized using the Cytofix/Cytoperm kit (eBioscience) in accordance with the manufacturer’s instructions. The cells were subsequently stained with antigranzyme B-AF647 (Thermo Fisher Scientific, clone GB11) in accordance with the manufacturer’s protocol. Following this staining, the fluorescence intensities of CD8 and granzyme B were analyzed using a FACSCalibur flow cytometer (BD Biosciences) and CellQuest software (BD Biosciences). To assess membrane PD-L1 expression, CRC cells were stained with anti-PD-L1 antibodies (Cell Signaling Technology, MA, USA), and median fluorescence intensity (MFI) was quantified by flow cytometry.

### T-cell transwell migration

T-cell migration assays were performed using established protocols with slight modifications [25]. Transwell inserts (Falcon, cat # 353,090) were placed in 24-well plates, and test media were added to the lower chambers. T-cells (1 × 10⁶) were seeded into the upper chambers and incubated for 24 h at 37 °C to facilitate migration. Each condition was tested in triplicate. The migrated T-cells were collected from the lower chambers and quantified using an automated cell counter (Bio-Rad, TC20).

### Pathway analysis

Pathway analyses were performed as previously described [26]. For each sample, gene set variation analysis (GSVA) was conducted using the corresponding R package to assess the levels of enrichment of predefined gene sets. The analysis focused on biological process categories from gene ontology gene sets obtained from the Molecular Signatures Database (MSigDB; https://www.gsea-msigdb.org/gsea/msigdb/). GSVA scores were computed for each sample to estimate the levels of enrichment of the gene sets obtained from the MSigDB, and correlations between the gene sets and CTSS mRNA expression were evaluated using Spearman’s correlation test. A Bonferroni-corrected *P* value (false discovery rate) threshold of 0.05 was applied.

### Monodansylcadaverine staining

Monodansylcadaverine (MDC) staining was employed to assess autophagy in CRC cells by labeling acidic vesicular organelles (AVOs), including lysosomes and autolysosomes. A previously reported method with minor modifications was employed [27]. Cells were seeded in 6-cm dishes and incubated overnight. The AVOs were then stained with 0.5 mM MDC (Sigma-Aldrich, # D4008) and incubated in phenol red-free RPMI at 37 °C for 30 min. The AVOs in the cells were visualized using a fluorescence microscope (Nikon, TE200). All experiments were conducted in triplicate.

### Ectopic expression of ptfLC3

The autophagy reporter plasmid ptfLC3 (encoding mRFP-GFP-MAP1LC3B; cat # 21,074; Addgene, Cambridge, MA, USA) was used to analyze autolysosomes through a previously described method [27]. Purified plasmids, obtained using the EndoFree Plasmid Mega Kit (Qiagen, 12,381), were transfected into CRC cells using Lipofectamine 3000 (Thermo Fisher Scientific, #L3000015). Briefly, 2 × 10^5^ CRC cells were seeded in 6-cm dishes and allowed to adhere overnight. The following day, purified plasmid DNA was diluted in Opti-MEM I medium without serum, mixed with the appropriate amount of P300 reagent, and incubated with diluted Lipofectamine 3000 reagent for 5 min at room temperature. The transfection mixture was then added to the cells and incubated for various durations.

### Immunofluorescent confocal microscopy

An immunofluorescence analysis was performed using a previously established protocol [24]. Briefly, HT29 cells were seeded onto glass coverslips and incubated for 24 h to allow adherence. The cells were then fixed with 4% paraformaldehyde at room temperature for 15 min, washed three times with ice-cold PBS, and permeabilized with PBST [PBS containing 1% Triton X-100 (Calbiochem, # 9410)] for 30 min. After permeabilization, the cells were blocked with solution containing 5% BSA (Sigma-Aldrich, # A2153) for 1 h at room temperature. The cells were subsequently incubated overnight at 4 °C with primary antibodies against PD-L1 (Cell Signaling, cat # 13,684) and LC3 (GeneTex, #GTX12375). Following three washes with TBST, the cells were incubated with a secondary antibody for 1 h at room temperature. After additional TBST washes, the slides were mounted using glycerol gelatin, and nuclei were counterstained blue with DAPI (Invitrogen, # P36935). Fluorescent images were acquired using a scanning confocal microscope (MPE, Olympus).

### Percoll gradient fractionation

Lysosomes were isolated using Percoll gradient fractionation, which was conducted using a previously described protocol [28]. Cells were pretreated with 5 μM CQ for 24 h, washed twice with PBS, and scraped into 2 mL of lysis buffer (comprising 10 mM ethanolamine, 1 mM EDTA, and 0.25 M sucrose; pH 7.2). The lysate was centrifuged at 800 × g for 5 min. The supernatant was collected, whereas the pellet was resuspended in 2 mL of homogenization buffer. After additional centrifugation at 4000 × g for 10 min, the supernatants were combined to obtain 4 mL of postnuclear supernatant. The postnuclear supernatant was mixed with 90% Percoll reagent (GE Healthcare; #17–0891-02) to achieve a final concentration of 30% Percoll in a total volume of 8 mL. The mixture was centrifuged at 28,000 × g for 24 h at 4 °C by using a Beckman SW28 rotor. The autolysosome-enriched fraction was carefully collected from the gradient without adjacent layers being disturbed, washed with cold PBS, and centrifuged at 20,000 × g for 30 min to remove excess Percoll. The final autolysosome fractions were analyzed through immunoblotting to detect PD-L1 and the lysosomal marker LAMP2.

### Establishment of orthotopic CRC model and tumor analysis

An orthotopic CRC model in immunocompetent mice was established as previously described [29]. All animal procedures were conducted in accordance with protocols approved by the Institutional Animal Care and Use Committee of the National Health Research Institutes (approval number: NHRI-IACUC-109164-A). Briefly, 1 × 10⁶ shCTSS or scramble MC-38 cells were injected into the rectum of C57BL/6 mice. After 4 weeks, the mice were euthanized, and rectal tumor tissues were excised and fixed in 10% formalin. Tumor dimensions were measured using a clipper, and tumor volume (*V*) was calculated as follows: *V* = 1/2*L* × *W* × *W*, where *L* represents tumor length and *W* represents tumor width. Tumor colonies were counted, and tissues were embedded in paraffin for sectioning into 4-μm sections for IHC staining. For IHC analysis, the tissue sections were subjected to antigen retrieval by autoclaving them in Tris–EDTA buffer (pH 9) at 121 °C for 10 min. The samples were then treated with 3% H₂O₂–methanol and incubated overnight at 4 °C with primary antibodies against CTSS, CD8 (PHARMINGEN, #555,367), CD4 (Thermo Fisher Scientific, # 14–0041-82), CD163 (Thermo Fisher Scientific, # BS-2527R), Granzyme B (Cell Signaling, #44,153), and PD-L1, each diluted 1:1,000 in antibody dilution buffer. Immunoreactive staining was visualized using the ab80436-EXPOSE Mouse and Rabbit Specific HRP/DAB Detection IHC Kit, and this was followed by hematoxylin counterstaining. For anti-CD8 treatment, 200 µg of anti-CD8 antibody (anti-mCD8-mIgG2a InvivoFit, #mcd8-mab10-1) was administered intraperitoneally every 7 days. The expression levels of CTSS, CD8, CD4, CD163, Granzyme B, and PD-L1 in the tumor tissues were scored as follows: 0 (no expression), 1 (0–25% positive tumor cells), 2 (26–50% positive tumor cells), and 3 (51–100% positive tumor cells).

### Statistical analysis

The Wilcoxon rank-sum test was employed to compare the mean of each group with that of the control group. All statistical analyses were two-sided, with a significance threshold of *P* < 0.05.

## Results

### Elevated CTSS expression is correlated with an immunosuppressive microenvironment and regulates PD-L1 expression in CRC

**Fig. 1:**
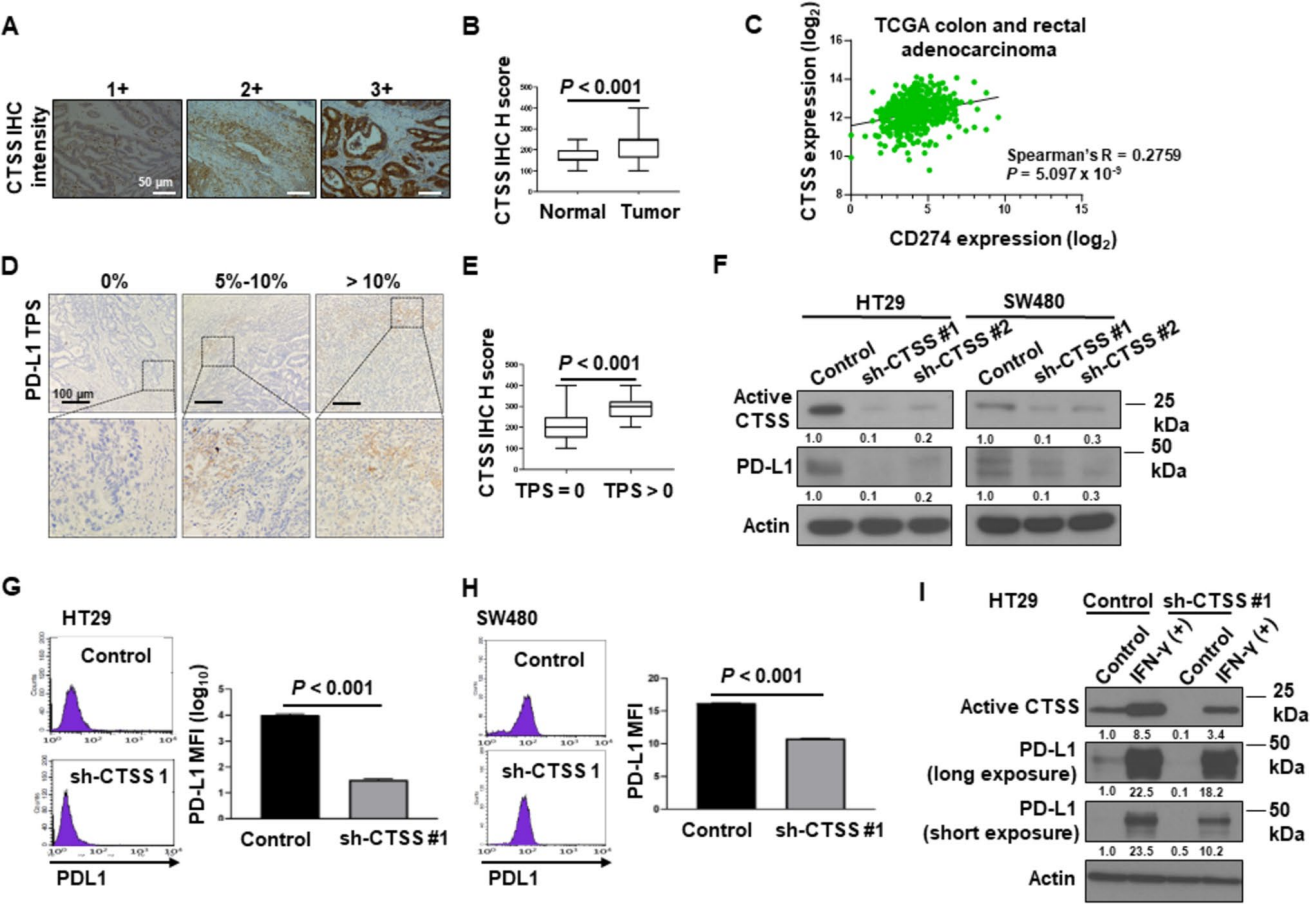
CTSS upregulation in CRC tissues. **A** Representative IHC images depicting CTSS expression scores of 1 + , 2 + , and 3 + in CRC tumor tissues. **B** Boxplots indicated that CTSS H-scores were higher in CRC tissues than in matched mucosa tissues. **C** Correlation of CTSS expression with immune checkpoint expression in CRC tissues, revealing a positive correlation between CTSS expression and PD-L1 levels. **D** Representative images of PD-L1 staining in CRC tumor cells with TPSs of 0%, 5%–10%, and > 10% shown at 100 × and 400 × magnification. **E** Boxplots revealing higher CTSS H-scores in CRC tissues with TPS > 0 than in those with TPS = 0. Scale bars and *P* values are indicated. Data are presented as mean ± standard deviation (SD) and were analyzed using the Wilcoxon rank-sum test. **F** Western blot analysis revealing lower PD-L1 levels in CTSS-deficient CRC cells compared with CTSS-proficient cells. **G** and **H** Flow cytometry histograms displaying membrane PD-L1 expression in HT29 **G** and SW480 **H** cells, with MFI reported as mean ± SD from three independent experiments. **I** Western blot analysis of PD-L1 expression in HT29 cells treated with 10 ng/mL IFN-γ for 24 h. Fold changes in PD-L1 levels were normalized to actin controls and quantified using ImageJ densitometry. *IHC* immunohistochemical; *MFI* median fluorescence intensity; *TPS* tumor proportion score

To investigate the role of CTSS in CRC progression, we assessed its expression in various cancer types. Transcriptomic data from the TCGA revealed that the mean CTSS expression was highest for CRC tissues among all evaluated cancer types (Supplementary Fig. 1A). Furthermore, CTSS expression was significantly elevated in CRC tissues compared with normal colon mucosa (Supplementary Fig. 1B). To examine the potential role of CTSS in MSS CRC, tumor tissues of patients were subjected to IHC staining (Fig. 1A, Table 1). Staining was successful in 92 samples, and both tumor tissues and adjacent mucosa were evaluable in 62 of these 92. H-score analysis demonstrated significantly higher CTSS expression in CRC tissues than in adjacent mucosa (Fig. 1B).

**Table 1:** Clinical and genomic characteristics of National Cheng Kung University Hospital cohort

Characteristics	Category	Case number
Sex	Male	46
	Female	46
Age (years)	< 60 years	52
	≥ 60 years	40
Primary tumor (T)	T1	5
	T2	6
	T3	66
	T4	15
Nodal status (N)	N1	64
	N2	28
Site	Cecum	11
	Ascending	4
	Transverse	7
	Descending	13
	Sigmoid	25
	Rectum	32
Histological grade	Mucinous	9
	Well differentiated	4
	Moderately differentiated Poorly differentiated	76 3
*RAS* mutation	Wild type Mutated	51 41

To evaluate the impact of CTSS on immune regulation in CRC, we analyzed its correlation with immune cell infiltration by using the TIMER database. In the TCGA colon adenocarcinoma dataset, CTSS expression was significantly negatively correlated with tumor purity (Rho = − 0.172, *P* = 0.0005) and significantly positively correlated with regulatory T-cells (Rho = 0.45, *P* = 4.13 × 10^−15^) and M2 macrophages (Rho = 0.426, *P* = 1.41 × 10^−13^; Supplementary Fig. 2A and B). Similar correlations were discovered in rectal adenocarcinoma, where CTSS expression was positively correlated with regulatory T-cells (Rho = 0.341, *P* = 1.01 × 10^−3^) and M2 macrophages (Rho = 0.314, *P* = 2.62 × 10^−3^; Supplementary Fig. 2C and D). These findings suggest that CTSS may promote an immunosuppressive microenvironment in CRC.

Given the critical role of immune checkpoint molecules in promoting an immunosuppressive microenvironment, we further investigated the correlation between CTSS expression and immune checkpoint gene expression by using TCGA transcriptome data. As shown in Fig. 1C and Supplementary Fig. 3A–D, CTSS expression was positively correlated with PD-L1 (CD274; Spearman’s R = 0.2759, *P* = 5.097 × 10^–9^), Tim3 (HAVCR2; Spearman’s R = 0.3111, *P* = 3.430 × 10^–11^), PD1 (PDCD1, Spearman’s R = 0.2338, *P* = 8.460 × 10^–7^), TIGIT (Spearman’s R = 0.3445, *P* = 1.537 × 10^–13^), and LAG3 (Spearman’s R = 0.3485, *P* = 7.808 × 10^–14^) in TCGA CRC datasets. Moreover, the TCGA data revealed a positive correlation between CTSS and PD-L1 expression in both non-MSS and MSS CRC patients (Supplementary Fig. 4). Given the clinical relevance of anti-PD-1/PD-L1-targeted therapies, we assessed PD-L1 expression in our CRC patient cohort. Tumors with high PD-L1 expression exhibited significantly higher CTSS H-scores compared with those with low PD-L1 expression (*P* < 0.001 for TPS > 0 vs. TPS = 0; Fig. 1D and E).

To determine whether CTSS directly regulates PD-L1 expression in CRC cells, we silenced CTSS by using shRNA in HT29 and SW480 CRC cells. In our initial screening (Supplementary Fig. 5), we assessed several CRC cell lines—including LoVo, HT29, SW480, and SW403—and selected HT29 and SW480 for downstream experiments due to their genetic diversity, efficient transfection performance, reproducible growth characteristics, and representative CTSS expression profiles. The results of the Western blot analysis revealed markedly lower PD-L1 expression in CTSS-deficient CRC cells compared with that in CTSS-proficient cells (Fig. 1F). Because PD-L1 on cancer cell membranes binds to PD-1 on T-cells, we next examined membrane PD-L1 expression in CTSS-proficient and CTSS-deficient CRC cells by performing flow cytometry. As illustrated in Fig. 1G and H, the MFI for membrane PD-L1 was lower in CTSS-deficient cells than in CTSS-proficient cells. Studies have demonstrated that IFN-γ secreted by CD8^+^ T-cells induces PD-L1 expression on tumor cells [30, 31]. Consistent with this, PD-L1 levels increased following 24-h IFN-γ treatment in HT29 cells in the present study; however, this upregulation was less pronounced in CTSS-deficient cells than it was in CTSS-proficient cells (Fig. 1I). These findings suggest that CTSS modulates PD-L1 expression in CRC cells both in the presence and absence of IFN-γ stimulation.

### CTSS-downregulated CRC cells promote cytotoxic T-cell activity

**Fig. 2:**
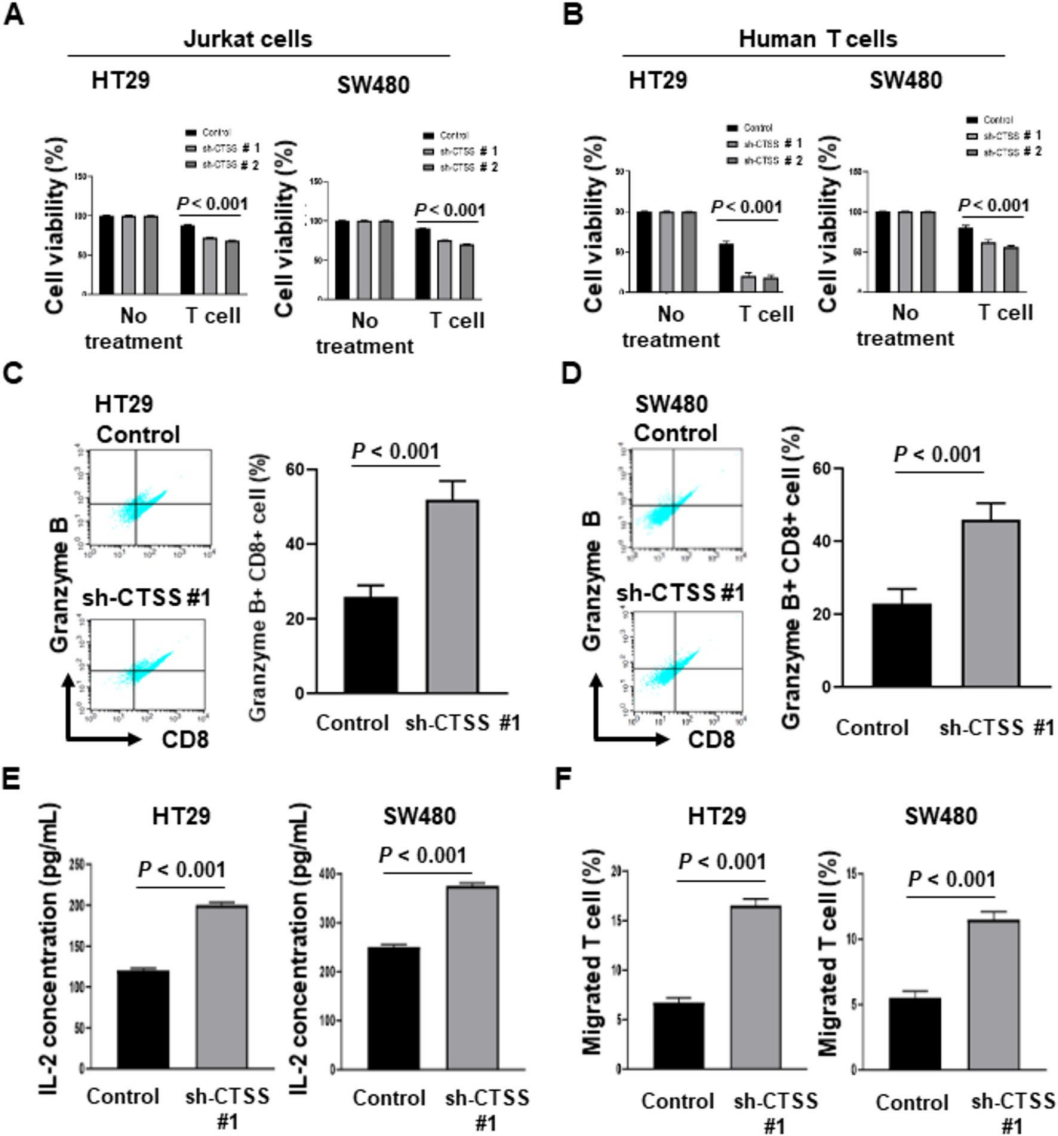
CTSS downregulation enhances cytotoxic T-cell activity against CRC cells. **A** Addition of activated Jurkat cells [effector cell to target cell (E:T) ratio of 20:1] enhanced the cytotoxicity of CTSS-deficient HT29 (left panel) and SW480 (right panel) compared with that of corresponding CTSS-proficient cells. Jurkat cells were activated through PHA treatment for the indicated duration. **B** Increased cytotoxicity was also observed in CTSS-deficient HT29 (left) and SW480 (right) cells following co-culture with human cytotoxic T-cells at an E:T ratio of 1:1. **C** and **D** Representative flow cytometry histograms of granzyme B expression in Jurkat cells cocultured for 24 h with CTSS-proficient or CTSS-deficient HT29 **C** and SW480 **D** cells by using a transwell system. Following incubation, T-cells were isolated, and granzyme B levels were assessed. **E** IL-2 concentrations in the conditioned media from Jurkat cells cocultured with HT29 (left panel) and SW480 (right panel) cells were measured using an ELISA kit after 24 h. **F** Transwell migration assays indicated higher T-lymphocyte migration toward conditioned media from CTSS-deficient HT29 (left panel) and SW480 (right panel) cells than for the controls. Migrated cells in the lower chamber were collected and quantified after 24 h. All experiments were performed in triplicate. *P* values are indicated

To assess whether downregulation of PD-L1 through CTSS suppression enhances T-cell-mediated cytotoxicity, CTSS-proficient and CTSS-deficient CRC cells were cocultured with activated Jurkat cells. Cytotoxicity assays revealed 15%–20% higher T-cell-induced cytotoxicity in CTSS-deficient CRC cells compared with the CTSS-proficient counterparts (Fig. 2A). When cocultured with human cytotoxic T-cells isolated from PBMCs, the CTSS-deficient CRC cells exhibited 20–40% higher cytotoxicity relative to CTSS-proficient cells (Fig. 2B). We next evaluated granzyme B expression levels in activated Jurkat cells cocultured with CRC cells. The percentage of granzyme B^+^ cytotoxic T-cells was 20%–30% higher for Jurkat cells cocultured with CTSS-deficient CRC cells than for those cocultured with CTSS-proficient cells (Fig. 2C and D). To determine whether CTSS modulates T-cell activation, we measured IL-2 concentrations in the conditioned media of activated Jurkat cells cocultured with CRC cells expressing different levels of CTSS. The IL-2 levels were 1.5- to 1.8-fold higher in media from T-cells cocultured with CTSS-deficient CRC cells than in media from T-cells cocultured with CTSS-proficient CRC cells (Fig. 2E). Finally, a transwell system was employed to investigate the role of CTSS in T-cell migration in the CRC microenvironment. Conditioned media from CTSS-deficient CRC cells resulted in 2.2- to 2.6-fold higher T-cell migration compared with media from CTSS-proficient cells (Fig. 2F). These findings collectively suggest that CTSS can modulate T-cell-mediated cytotoxicity in CRC.

### CTSS suppression activates autophagy in CRC cells

**Fig. 3:**
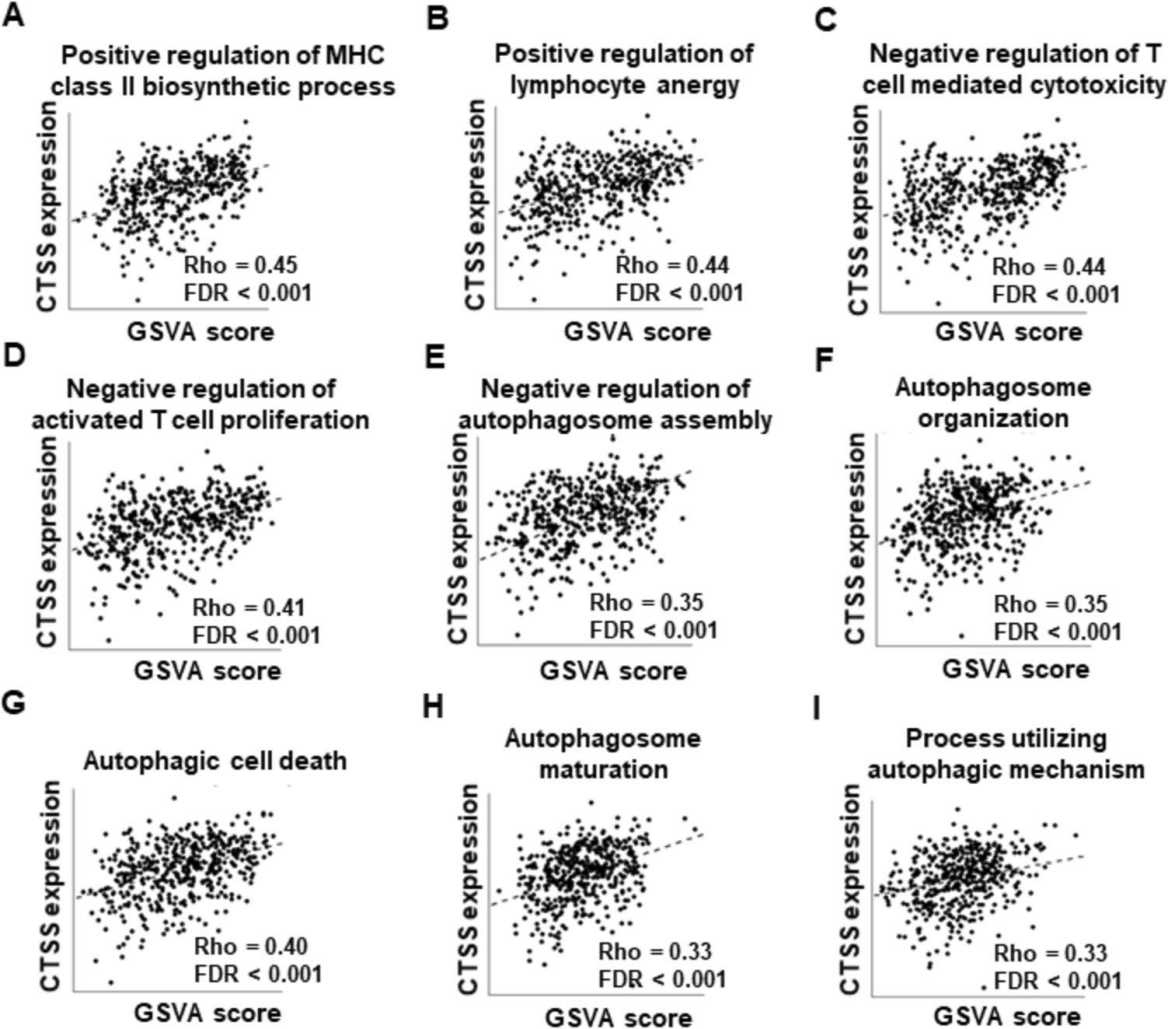
GSVA reveals CTSS-associated biological processes in the CRC TME. Immune-related cell functions associated with CTSS expression included the following: **A** positive regulation of MHC class II biosynthetic process, **B** positive regulation of lymphocyte anergy, **C** negative regulation of T-cell-mediated cytotoxicity, and **D** negative regulation of activated T-cell proliferation. Autophagy-related functions associated with CTSS expression included the following: **E** negative regulation of autophagosome assembly, **F** autophagosome organization, **G** autophagic cell death, **H** autophagosome maturation, and **I** processes involving autophagic mechanisms. Spearman correlation coefficients and false discovery rate (FDR) values are indicated. *GSVA* gene set variation analysis; *MHC* major histocompatibility complex

To investigate the biological processes mediated by CTSS in the CRC microenvironment, we performed GSVA by using TCGA data (Supplementary Table 1). Consistent with previous findings [17], we found that CTSS expression was positively associated with the regulation of MHC class II biosynthesis, suggesting an involvement of CTSS in CRC immunity (Fig. 3A). Additionally, CTSS expression was correlated with immune-regulatory pathways, including the positive regulation of lymphocyte anergy, negative regulation of T-cell-mediated cytotoxicity, and negative regulation of activated T-cell proliferation (Fig. 3B–D). These findings are consistent with our in vitro observations of CTSS-mediated modulation of T-cell activity in CRC cells. In addition to the IFNγ/JAK/STAT signaling pathway, autophagy has been implicated in the regulation of PD-L1 expression in cancer cells [32]. In our analysis, CTSS expression was negatively associated with several autophagy-related pathways, including autophagosome assembly, autophagosome organization, autophagic cell death, autophagosome maturation, and processes involving autophagic mechanisms (Fig. 3E–3I). Taken together, these findings suggest that CTSS significantly influences both cytotoxic T-cell activation and autophagy regulation in the CRC microenvironment.

### CTSS regulates autophagy flux in CRC cells

**Fig. 4:**
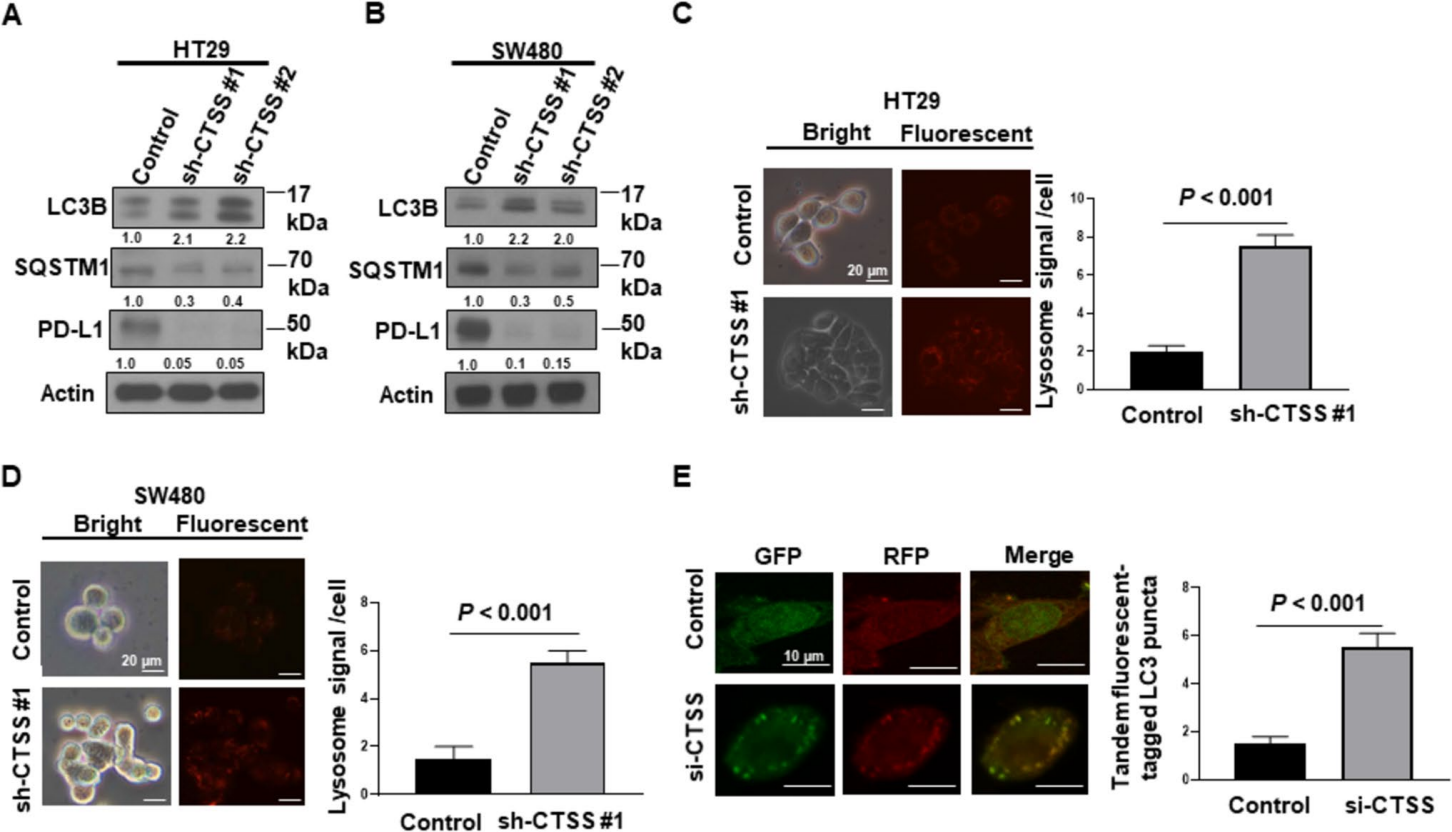
CTSS suppression induces autophagy activation. **A** HT29 and **B** SW480 cells with various levels of CTSS expression (control, shCTSS 1, and shCTSS 2) were cultured for 48 h, after which proteins were extracted. Western blot analysis was performed to examine the expression levels of LC3B, SQSTM1, and PD-L1. **C** HT29 and **D** SW480 cells with various levels of CTSS expression were seeded in six-well plates (1 × 10^5^ cells/well). After 3 days, the cells were incubated with LysoTracker Deep Red probe for 30 min at 37 °C, and lysosomal activity was visualized using fluorescence microscopy. **E** HT29 parental cells were seeded in six-well plates (2 × 10^5^ cells/well). After 24 h, the cells were transfected with the ptfLC3 plasmid. Following an additional 24 h, the cells were transfected with si-CTSS and maintained for 48 h, and images were taken using fluorescence microscopy. Protein expression fold changes were normalized to actin and quantified using ImageJ densitometry. *P* values are indicated. *GFP* green fluorescent protein; *RFP* red fluorescent protein

Studies have demonstrated that CTSS inhibition induces autophagy in various cancers, including lung, oral, and glioma cancers [28, 33, 34]. To investigate the relationship between CTSS expression and autophagy activation in CRC cells, we evaluated the expression of LC3B and SQSTM1 in CTSS-proficient and CTSS-deficient cells. Western blot analysis revealed that CTSS suppression enhanced LC3B-II conversion and reduced SQSTM1 expression in HT29 and SW480 cells (Fig. 4A and B). Fluorescence microscopy further confirmed more numerous acidic vesicular organelles—indicative of autolysosomes and lysosomes—in CTSS-deficient HT29 and SW480 cells (Fig. 4C and D). Quantitative analyses revealed 3.5- to 3.7-fold higher lysosomal signal intensity in CTSS-deficient cells compared with CTSS-proficient controls. Moreover, in ptfLC3-transfected HT29 cells, CTSS knockdown via siRNA led to an increase in the number of both yellow (autophagosomes) and red (autolysosomes) LC3 puncta (Fig. 4E). A quantitative analysis indicated an approximately 4.0-fold greater number of yellow and red puncta in CTSS-deficient cells compared with CTSS-proficient cells. These findings suggest that CTSS is essential for regulating autophagy flux in CRC cells.

### CTSS downregulation suppresses PD-L1 expression through autophagy in CRC cells

**Fig. 5:**
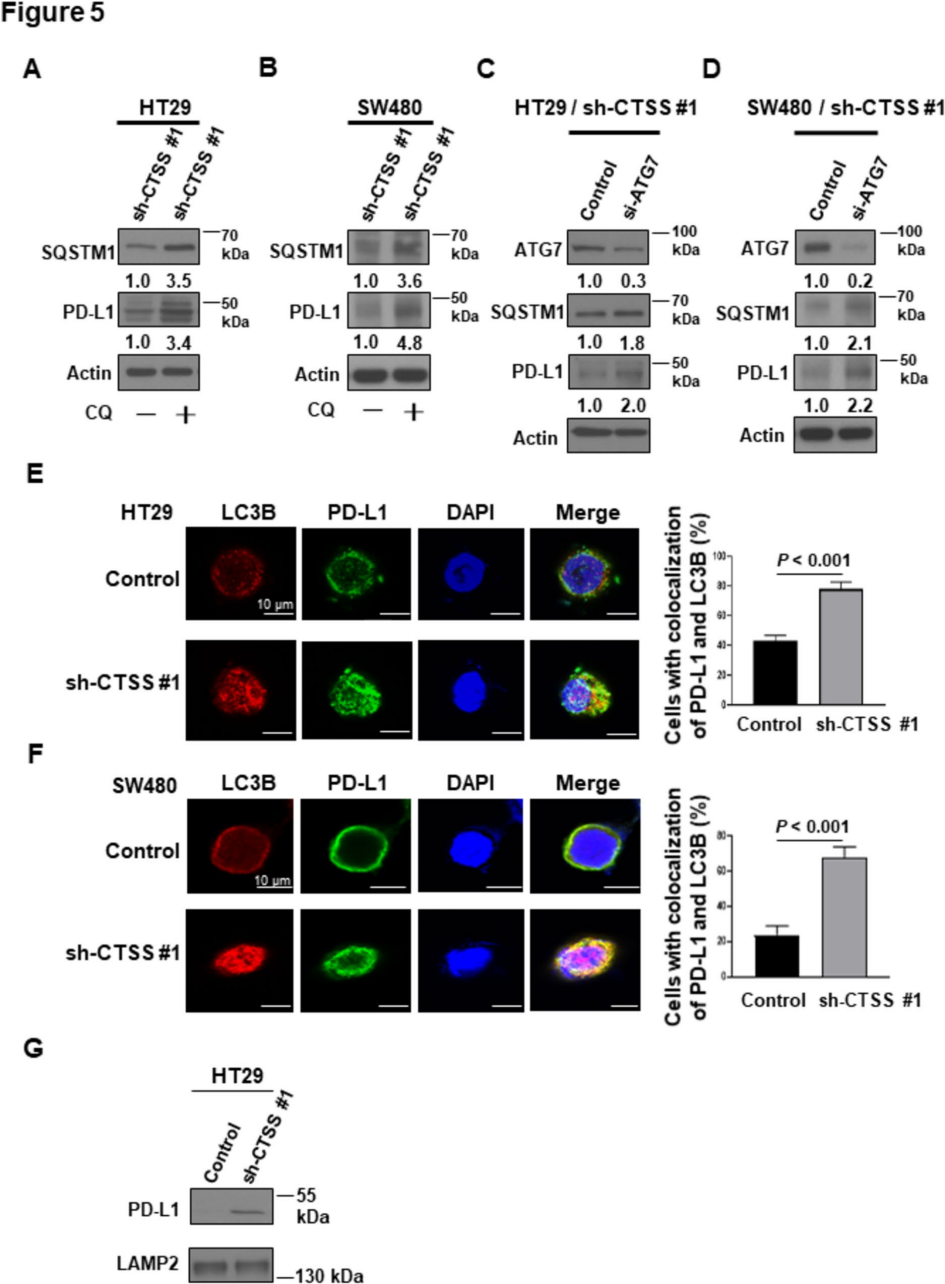
CTSS downregulation suppresses PD-L1 expression through autophagy in CRC cells. **A** HT29 and **B** SW480 cells treated with sh-CTSS1 were seeded in six-well plates (2 × 10^5^ cells/well). After 24 h, the cells were treated with 5 μM chloroquine (CQ) for 48 h. Protein lysates were analyzed through Western blot analysis of SQSTM1 and PDL-1 expression. **C** HT29 cells and **D** SW480 cells treated with sh-CTSS1 were seeded in six-well plates (2 × 10^5^ cells/well). After 24 h, the cells were transfected with si-ATG7 for 48 or 72 h. Thereafter, proteins were collected, and Western blot analysis was performed to assess ATG7, SQSTM1, and PDL-1 expression. **E** HT29 and **F** SW480 cells (control and sh-CTSS1) were seeded in chamber slides (7000 cells/well). After 24 h, the cells were treated with 5 μM CQ for 24 h. Confocal microscopy was used to evaluate the colocalization of LC3B and PDL-1. **G** HT29 cells (control and sh-CTSS1) were seeded in 15-cm dishes until 80% confluency. Subsequently, they were treated with 5 μM CQ for 24 h. The cells were then harvested, and lysosomes were isolated through ultracentrifugation. Western blot analysis was performed to assess PD-L1 and LAMP2 expression in the lysosomal fractions. Protein expression fold changes were normalized to actin and quantified using ImageJ densitometry. *P* values are indicated. *CQ* chloroquine

To investigate the mechanisms underlying CTSS-mediated PD-L1 downregulation, we evaluated PD-L1 expression in CTSS-deficient CRC cells following their treatment with CQ, an autophagy inhibitor. As shown in Fig. 5A and B, CQ treatment led to an increase in SQSTM1 levels and mitigated the reduction in PD-L1 expression induced by CTSS suppression. Furthermore, flow cytometry analysis demonstrated that CQ treatment increased membrane PD-L1 expression in CTSS-deficient HT29 cells compared to untreated cells (Supplementary Fig. 6). Given the role of *ATG7* in autophagosome formation, we employed siRNA to silence *ATG7* in CTSS-deficient CRC cells. *ATG7* knockdown enhanced the expression of both SQSTM1 and PD-L1 in CTSS-deficient cells (Fig. 5C and D). To confirm that CTSS suppression promotes PD-L1 degradation through an autophagy-related pathway, we examined the colocalization of LC3 and PD-L1 in CRC cells by using immunofluorescent confocal microscopy. As shown in Fig. 5E and F, the number of PD-L1-LC3 colocalization foci was markedly higher in CTSS-deficient cells than in CTSS-proficient controls. A quantitative analysis revealed a 2.0- to 2.8-fold higher proportion of cells exhibiting colocalization of PD-L1 and LC3B in CTSS-deficient CRC cells. Additionally, lysosomal fractions isolated from HT29 cells exhibited higher PD-L1 levels in CTSS-deficient cells than in their CTSS-proficient counterparts (Fig. 5G). These findings collectively indicate that CTSS suppression reduces PD-L1 expression through an autophagy-dependent pathway in CRC cells.

### CTSS suppression inhibits tumor growth and enhances T-cell activation in MC38 mouse model

**Fig. 6:**
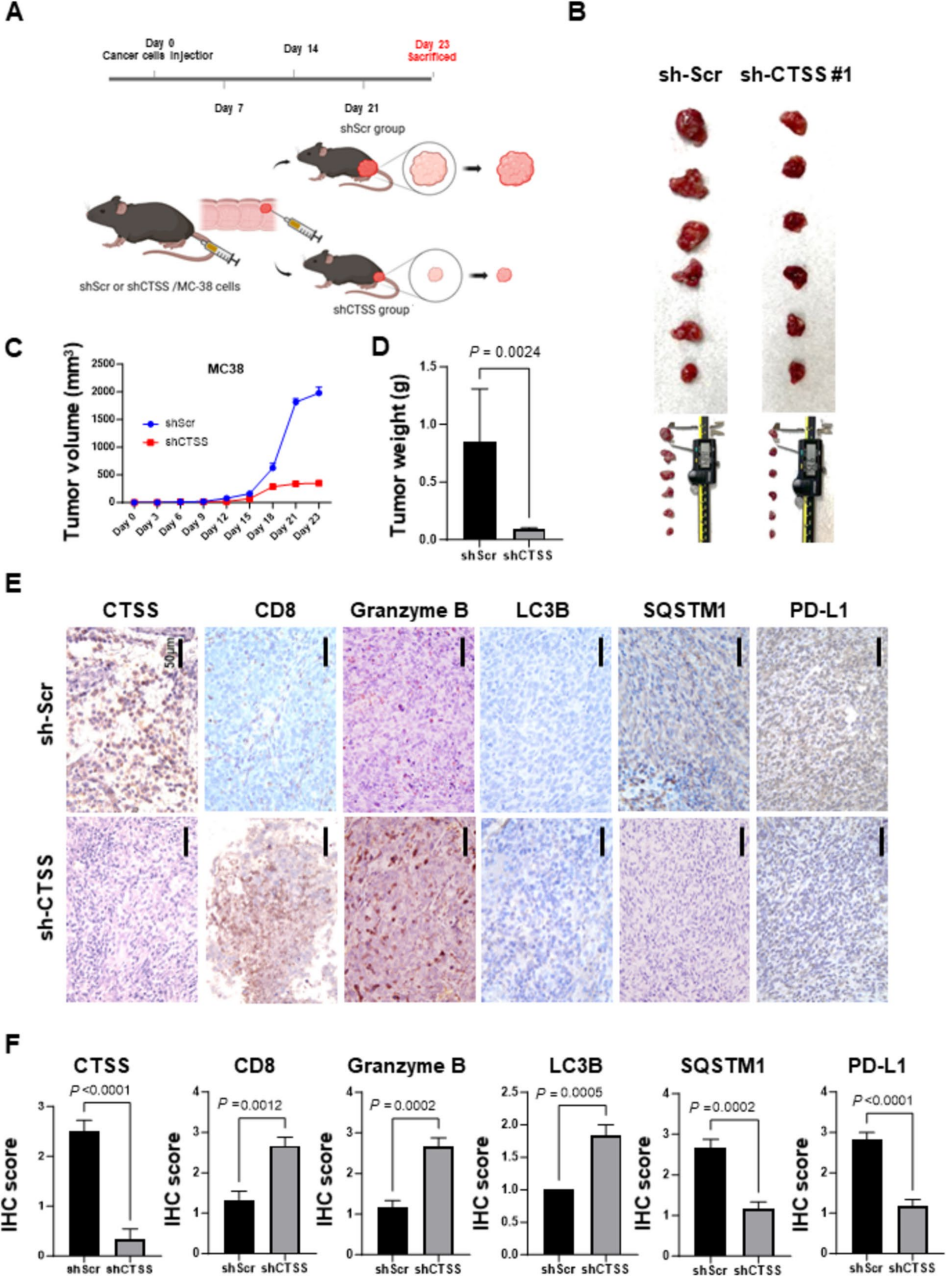
CTSS suppression inhibits MC38 tumor growth in immunocompetent mice. **A** Schematic of the establishment of an orthotopic CRC mouse model. The mice were sacrificed 4 weeks after MC38 cell injection. **B** Representative images of tumors excised from C57BL/6 mice orthotopically injected with MC38 cells expressing either shCTSS or shScr. **C** Tumor volume was measured every 3 days, and **D** tumor weight was recorded upon rectal excision. **E** Representative IHC images of tumor tissues stained with antibodies against CTSS, CD8, SQSTM1, LC3B, granzyme B, and PD-L1. Scale bar: 50 μm. **F** Quantitative IHC analysis of tumor tissues from shScr and shCTSS groups (n = 12). Tumor cell staining was scored as follows: 0 = no expression, 1 = 1–25% positive cells, 2 = 26–50% positive cells, and 3 = 51–100% positive cells. Data are presented as mean ± SD and were analyzed using a two-tailed Student’s *t* test. *P* values are indicated. *g* gram; *IHC* immunohistochemical; shScr, shScramble

On the basis of our in vitro findings, we hypothesized that CTSS expression influences CRC tumor growth through the modulation of tumor immunity. To test this hypothesis in vivo, we orthotopically injected CTSS-deficient and CTSS-proficient MC-38 cells into the rectum of C57BL/6 mice. After 4 weeks, the mice were euthanized, and tumor weight and size were assessed (Fig. 6A and B). The results indicated that tumors derived from CTSS-deficient cells were significantly smaller and lighter than those from CTSS-proficient controls (Fig. 6C and D). IHC analysis revealed significantly more infiltration of CD8^+^ T-cells and higher granzyme B expression in the CTSS-deficient tumors (Fig. 6E and F). To further investigate CTSS-mediated tumor immunity, we assessed CD4⁺ T-cell and M2 macrophage (CD163⁺ cells) infiltration in MC38 tumors. As shown in Supplementary Fig. 7, IHC analysis revealed a significant increase in CD4⁺ T-cell infiltration in CTSS-deficient tumors. Although the reduction in M2 macrophages did not reach statistical significance, a trend toward decreased CD163⁺ cell presence was observed. These findings suggest that CTSS may modulate not only T-lymphocyte-mediated immunity but also immunosuppressive myeloid populations. Additionally, autophagy markers were altered in these tumors, with SQSTM1 expression being lower and LC3B expression being higher, consistent with enhanced autophagy activity. Notably, PD-L1 expression was significantly lower in CTSS-deficient tumors. Collectively, these findings suggest that CTSS plays a critical role in regulating CRC immunity by mediating PD-L1 degradation through autophagy-related mechanisms.

### Anti-CD8 antibody treatment promotes tumor growth in CTSS-proficient MC38 mouse model

**Fig. 7:**
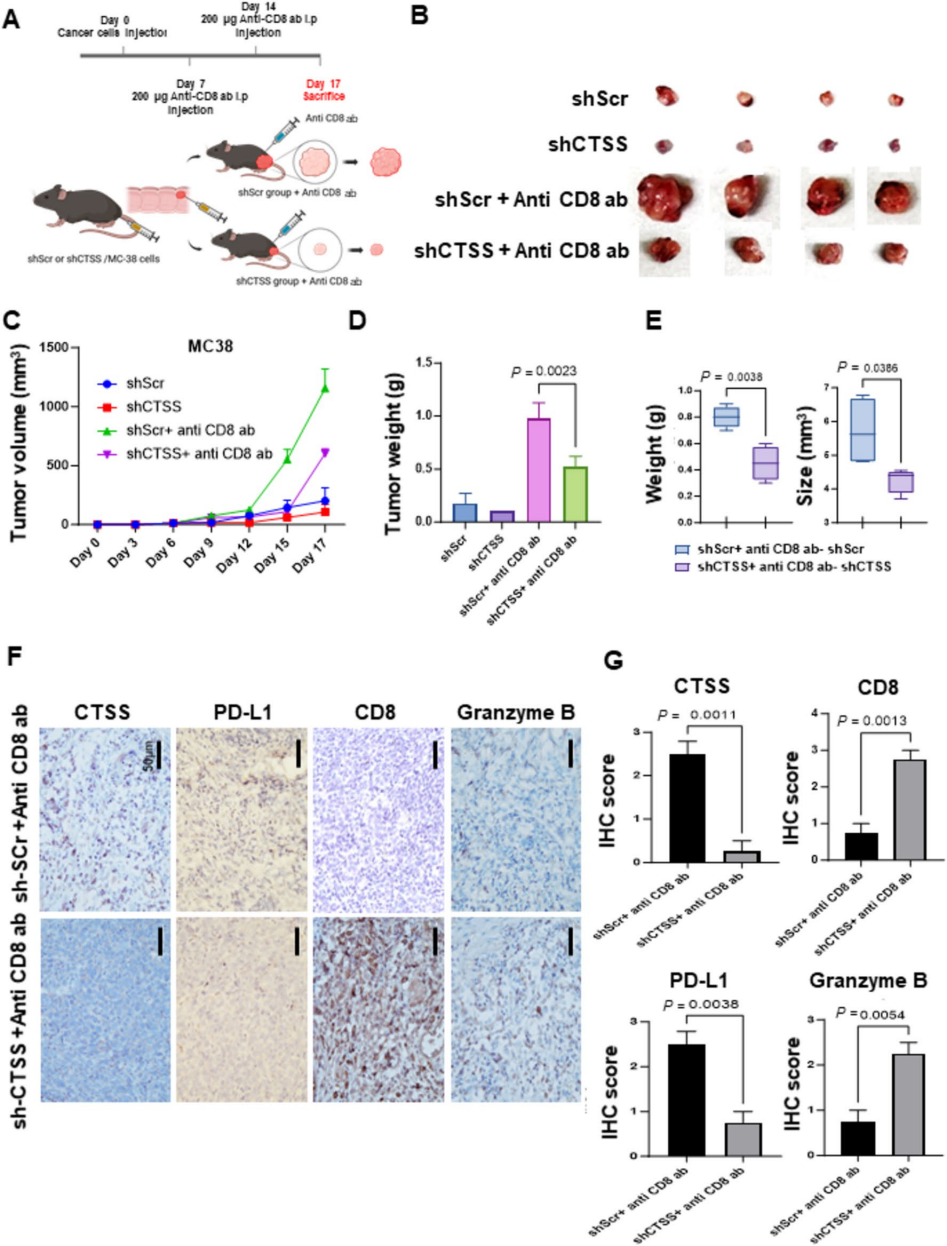
Effects of CD8 depletion on tumor growth in mice injected with control or CTSS-suppressed MC38 cells. **A** Schematic of the establishment of the orthotopic CRC mouse model. Anti-CD8 antibodies were administered intraperitoneally on days 7 and 14. **B** Representative images of tumors excised from C57BL/6 mice orthotopically injected with MC38 cells expressing either shCTSS or shScramble, with or without anti-CD8 treatment. **C** Tumor volume was measured every 3 days, and **D** tumor weight was recorded after rectal excision. **E** The increase in tumor weight induced by anti-CD8 antibody treatment was compared for the shScr group versus shCTSS group. **F** Representative IHC images of tumor tissues stained with antibodies against CTSS, PD-L1, CD8, and granzyme B. Scale bar: 50 μm. **G** Quantitative IHC analysis of tumor tissues from shScramble and shCTSS groups treated with anti-CD8 antibodies. Tumor cell staining was scored as follows: 0 = no expression, 1 = 1–25% positive cells, 2 = 26–50% positive cells, and 3 = 51–100% positive cells. Data are represented as mean ± SD and were analyzed using a two-tailed Student’s *t* test. *P* values are indicated. *ab* antibody; *g* gram; *IHC* immunohistochemical; *shScr* shScramble

**Fig. 8:**
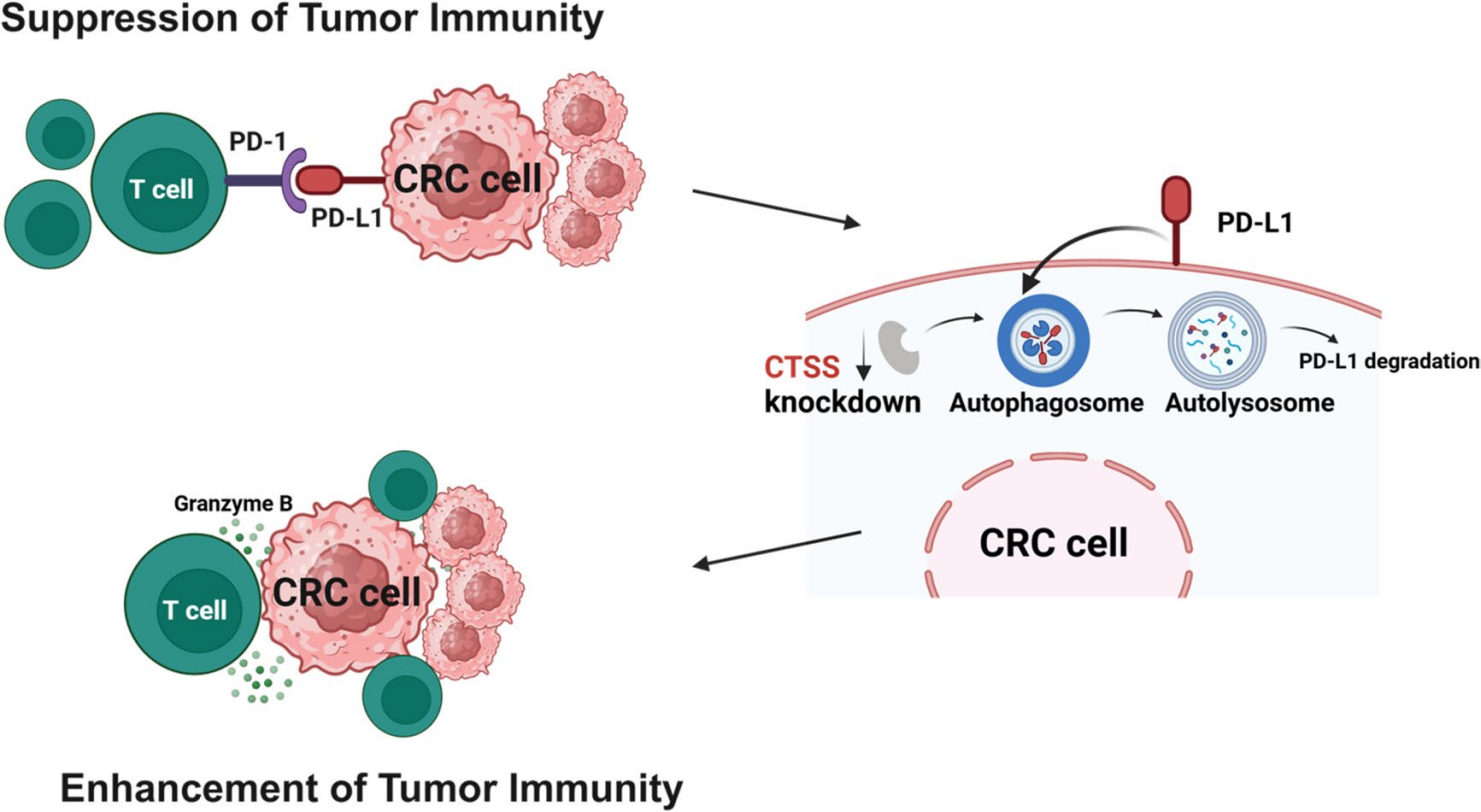
Schematic of the proposed mechanism through which CTSS depletion induces autophagy-associated PD-L1 degradation in CRC cells and enhances T-cell-mediated cytotoxicity against tumor cells. *CRC* colorectal cancer

To evaluate the role of CD8^+^ T-cells in CTSS-mediated tumor immunity, we administered anti-CD8 antibodies (Anti-mCD8-mIgG2a InvivoFit) in an orthotopic CRC mouse model. Two weeks after this treatment, the mice were euthanized, and tumor size and weight were assessed (Fig. 7A–D). Tumors derived from shCTSS or shCTSS + anti-CD8 cells were significantly smaller and lighter than those in their respective controls. Notably, tumor weight increased significantly more in the control group than in the CTSS-suppressed cells (Fig. 7E). IHC staining revealed elevated CD8^+^ T-cell infiltration and increased granzyme B expression in shCTSS + anti-CD8 tumors, suggesting enhanced CD8^+^ T-cell activity in CTSS-suppressed CRC cells compared with that in CTSS-proficient cells (Fig. 7F and G). These findings underscore the pivotal role of CTSS in modulating cytotoxic T-cell responses within the CRC TME by regulating PD-L1 expression through autophagy-related mechanisms (Fig. 8).

## Discussion

CRC remains one of the most prevalent malignancies worldwide, underscoring the urgent need for effective therapeutic strategies. Although ICIs have been demonstrated to have clinical benefit in some malignancies, their efficacy in advanced CRC has been limited, and this emphasizes the importance of elucidating tumor immune-regulatory mechanisms. In this study, we identified a positive correlation between CTSS and PD-L1 expression in CRC tissues and cell lines. Functional assays revealed that CTSS suppression enhanced T-cell activation and cytotoxicity against CRC cells, implicating CTSS as a key contributor to the immunosuppressive TME and a potential immunotherapeutic target in CRC. Mechanistically, this is the first study to demonstrate that CTSS suppression promotes autophagy and that inhibition of autophagy restores PD-L1 expression in CTSS-deficient CRC cells. These findings suggest that CTSS regulates PD-L1 expression by modulating autophagic flux, offering novel insights into the interplay between autophagy and immune checkpoint regulation in CRC cells.

CTSS, known for its inflammation-modulating properties, is implicated in cardiovascular, neurological, renal, immunological, and malignant conditions [17, 35–37]. In some cancer types, CTSS contributes to tumor progression through multifaceted mechanisms [18–22]. Although early studies have emphasized its role in promoting invasion and angiogenesis, more recent evidence highlights additional roles, including the promotion of a protumorigenic microenvironment, modulation of key signaling pathways, and facilitation of metastasis. CTSS is produced not only by tumor cells but also by various stromal components within the TME, including endothelial and infiltrating immune cells [20–22]. Its proteolytic activity is associated with MHC II antigen presentation and the recruitment of tumor-associated macrophages in the TME, further implicating CTSS in the regulation of antitumor immune responses. Elevated CTSS expression has been associated with CRC with aggressive clinical features, including higher histological grade, advanced clinical stage, and increased recurrence risk [19, 38]. Consistent with these findings, the present study demonstrated significantly higher CTSS expression in MSS CRC tissues compared with adjacent normal tissues (Fig. 1A and B). Furthermore, public dataset analyses revealed positive correlations of CTSS expression with immunosuppressive cell infiltration as well as with key immune checkpoint molecules in CRC tissues (Supplementary Fig. 2 and 3). These findings underscore the pivotal role of CTSS in modulating the immunological landscape of CRC.

Cancer cells often express PD-L1, which binds to PD-1 receptors on immune cells, particularly T-cells, thereby suppressing their antitumor activity and facilitating immune evasion [13, 14]. Although activation of the PD-1/PD-L1 axis is associated with poorer outcomes in advanced cancer stages, high PD-L1 expression also renders tumor cells more sensitive to PD-1/PD-L1 inhibitors. These inhibitors disrupt the PD-1/PD-L1 interaction, thereby restoring T-cell-mediated immune surveillance and cytotoxicity. Microsatellites are repetitive DNA sequences scattered throughout the genome [7, 8]. Defects in the DNA mismatch repair system, which typically corrects replication errors, lead to MSI. MSI status is a critical prognostic and predictive biomarker in CRC, particularly for immunotherapy responses. However, only approximately 5% of metastatic CRC cases exhibit MSI-H, limiting the eligibility of the majority of patients with advanced CRC for ICI treatment [39]. Expanding the clinical utility of PD-1/PD-L1 inhibitors thus requires a deeper understanding of the regulatory mechanisms controlling PD-L1 expression in CRC. In the current study, CTSS expression was strongly associated with PD-L1 levels in CRC tissues (Fig. 1C–E). In CRC cell lines, CTSS deficiency significantly reduced PD-L1 expression at both the total protein and membrane levels (Fig. 1G–H). PD-L1 expression can also be upregulated in response to IFN-γ released by T-cells upon recognition of tumor neoantigens presented through MHCs. Although this response enhances immune recognition, it also facilitates immune evasion [30, 31]. Notably, IFN-γ-induced PD-L1 upregulation was significantly reduced in CTSS-deficient CRC cells (Fig. 1I). These results suggest that CTSS not only regulates baseline PD-L1 expression but also influences PD-L1 expression in CRC cells in response to IFN-γ stimulation.

To assess the functional effect of CTSS-mediated PD-L1 expression on CRC immunity, we performed coculture assays by using activated Jurkat cells and T-cells isolated from PBMCs. CTSS-deficient CRC cells exhibited significantly worse survival compared with CTSS-proficient cells (Fig. 2A and B). Cytotoxic T-cells cocultured with CTSS-deficient CRC cells exhibited elevated levels of granzyme B, a serine protease essential for T and NK cell-mediated cytotoxicity [40] (Fig. 2C and D). Furthermore, IL-2, a cytokine critical for T-cell proliferation and function [41], was significantly elevated in the conditioned medium from T-cells cocultured with CTSS-deficient CRC cells (Fig. 2E). This was accompanied by enhanced T-cell migration toward conditioned medium derived from CTSS-deficient CRC cells (Fig. 2F). These findings collectively indicate that CTSS modulates PD-L1 expression and influences T-cell-mediated immunity responses against CRC cells. To support our in vitro findings with clinical evidence, we analyzed data from public datasets. GSVA of the TCGA dataset revealed a positive association between CTSS expression and the MHC class II biosynthetic process, a known function of CTSS [17]. Additionally, the GSVA data indicated the involvement of CTSS in immunosuppressive processes within the CRC microenvironment, including positive regulation of lymphocyte anergy, negative regulation of T-cell-mediated cytotoxicity, and negative regulation of activated T-cell proliferation (Fig. 3). Together, these bioinformatic and experimental findings underscore the critical role of CTSS in shaping the immunosuppressive microenvironment of CRC by modulating PD-L1 expression and T-cell activity.

The regulation of PD-L1 expression in cancer cells is multifaceted and involves contributions from tumor cells, immune cells, and stromal components within the TME [42, 43]. Autophagy, a catabolic process essential for maintaining cellular homeostasis, facilitates the degradation of intracellular components such as proteins, lipids, and mitochondria within lysosomes. Although some studies have reported conflicting results, increasing evidence supports a role for autophagy in degrading PD-L1, thereby enhancing T-cell activity and promoting tumor suppression, including in CRC cells [44–46]. In inflammatory conditions such as cardiovascular disorders and chronic obstructive pulmonary disease, CTSS inhibition has been shown to induce autophagy [17, 47, 48]. However, the interplay between CTSS and autophagy in cancer cells remains underexplored [34]. In this study, GSVA analyses revealed a correlation between CTSS expression and autophagy-related pathways in CRC tissues (Fig. 3). To further investigate this relationship, we examined autophagy markers in CRC cells. CTSS suppression induced autophagy, as evidenced by reduced SQSTM1 levels and increased LC3B-II conversion. Immunofluorescence analysis corroborated these findings, revealing enhanced MDC staining and LC3 puncta formation in autolysosomes (ptfLC3 plasmid) in CTSS-deficient CRC cells (Fig. 4). Moreover, inhibition of autophagy by using CQ or ATG7-targeting siRNA restored PD-L1 expression in CTSS-deficient CRC cells. Increased colocalization of PD-L1 with LC3, along with elevated PD-L1 levels in lysosomal fractions, further supports the role of CTSS in regulating PD-L1 degradation through autophagic flux (Fig. 5). Animal models have long been pivotal in advancing CRC research because they provide insights into tumor biology and interactions with the microenvironment [49, 50]. Therefore, we established an orthotopic CRC mouse model by injecting MC-38 cells into the rectum of C57BL/6 mice to mimic the interactions between CRC and its TME (Fig. 6). In this in vivo model, CTSS knockdown resulted in increased infiltration of CD8^+^ T-cells expressing granzyme B along with enhanced autophagy markers and reduced PD-L1 expression in tumor cells. Moreover, treatment with anti-CD8 antibodies led to a more pronounced increase in tumor weight in CTSS-proficient cells compared with that in CTSS-deficient cells, further supporting the role of CTSS in modulating cytotoxic T-cell function (Fig. 7). Collectively, these findings establish CTSS as a key regulator of immune dynamics within the CRC TME. By regulating PD-L1 expression through autophagy, CTSS is a promising immunotherapeutic target for CRC (Fig. 8).

Researchers have pursued the clinical development of CTSS inhibitors for decades, with the initial target being autoimmune diseases [47, 51, 52]. Early research efforts prioritized inhibitor potency, leading to compounds that lacked specificity for CTSS and exhibited off-target activity against other cysteine cathepsins [52]. This lack of selectivity may have contributed to the failure of initial clinical trials. Recent research has prioritized the design of CTSS inhibitors that are both potent and highly specific. This approach involves targeting key amino acid residues in the S2 and S3 pockets of CTSS, which are critical for achieving specificity [52]. Advances in molecular modeling have facilitated this process, resulting in the identification of promising inhibitors such as LY3000328 and RO5459072, both of which have progressed to clinical trials for immunogenic and cardiovascular conditions [53, 54]. Additionally, preclinical studies by Chen et al. demonstrated that a specific CTSS inhibitor, RJW-58, can mitigate peripheral neuropathy through immunomodulatory mechanisms [55]. Given the limited efficacy of current immunotherapies in CRC, exploring combination strategies that integrate specific CTSS inhibitors with existing immunotherapeutic agents is essential. Such approaches could enhance therapeutic efficacy and address unmet clinical needs in CRC. In parallel, the field of autophagy modulation in cancer therapy is rapidly evolving, particularly in the context of combinatorial treatment strategies [56–58]. Several ongoing clinical trials are investigating the use of autophagy modulators, such as CQ and mTOR inhibitors, in combination with immunotherapies [58–60]. Our findings indicate that CTSS-mediated autophagy significantly influences tumor immunity within the CRC microenvironment. Despite the inherent complexity of autophagy and its interplay with the TME, targeting autophagy in conjunction with immunotherapy is a promising strategy. This combined approach may offer a novel therapeutic avenue for enhancing treatment outcomes in patients with CRC.

This study has several limitations that warrant further investigation. One important limitation is the lack of data linking CTSS expression to post-translational modifications (PTMs) or isoforms of PD-L1. The mechanisms regulating tumor PD-L1 expression are complex and involve both intrinsic and extrinsic pathways. In addition to IFN-γ-induced signaling, a range of PTMs—including glycosylation, phosphorylation, ubiquitination, and acetylation—have been shown to modulate PD-L1 stability, localization, and function [61–63]. These modifications play a critical role in shaping the dynamics of PD-1/PD-L1 interactions and, consequently, tumor immune evasion. Moreover, distinct PD-L1 isoforms may possess divergent biological functions [64–66]. For example, nuclear PD-L1 has been shown to interact with the cohesin complex to regulate sister chromatid cohesion, while exosome-associated PD-L1 (exo-PD-L1) contributes to immunosuppressive signaling and modulates the tumor microenvironment through intercellular communication. Similarly, soluble PD-L1 (sPD-L1) may act as a decoy receptor to suppress T-cell activity. In our study, we observed two PD-L1 bands of differing molecular weights in HT29 and SW480 cells, especially upon longer immunoblot exposure, suggesting the presence of distinct glycoforms or isoforms. Given that our current focus was on elucidating the role of CTSS-mediated autophagy in regulating PD-L1 expression, we did not investigate whether CTSS also contributes to PD-L1 PTMs or splicing alterations. This remains an important area for future exploration to better understand the multifaceted regulatory mechanisms controlling PD-L1 in CRC.

Another important consideration is the potential role of CTSS expression as a predictive biomarker for anti-PD-L1/PD-1 therapies. Given that PD-L1 expression is widely used to predict response to immune checkpoint blockade, the observed positive correlation between CTSS and PD-L1 expression in our study raises the possibility that CTSS may have predictive value as well. Future clinical studies are warranted to explore the potential utility of CTSS expression as a companion biomarker for guiding anti-PD-L1/PD-1 immunotherapy in CRC and potentially other malignancies. Beyond PD-L1, additional immune checkpoint molecules—including TIM-3, TIGIT, and LAG-3—have emerged as promising therapeutic targets, with encouraging findings in both preclinical models and clinical trials [67, 68]. In our analysis of TCGA CRC datasets, CTSS expression was positively correlated with the expression of these alternative immune checkpoints (Supplementary Fig. 3), suggesting that CTSS may be associated with broader immunosuppressive mechanisms. These results highlight the need for future investigations to assess whether CTSS inhibition can modulate the expression of multiple immune checkpoints and whether combinatorial approaches—such as CTSS inhibition in combination with anti-TIGIT or anti-LAG3 therapies—could enhance and sustain antitumor immune responses.

Lastly, an important question is whether CTSS inhibition could also benefit MSI-H CRC patients, who are generally more responsive to ICIs [39]. As noted, our study primarily focused on MSS CRC, which represents the majority of CRC cases and is typically resistant to current immunotherapy approaches [39]. However, our bioinformatic analysis revealed that CTSS is also expressed in MSI-H CRC and shows a positive correlation with PD-L1 expression (Supplementary Fig. 4). These findings suggest that CTSS-targeted strategies may be relevant across a broader CRC patient population, encompassing both MSS and MSI-H subtypes. Nevertheless, the therapeutic implications may be particularly significant for MSS CRC, where the efficacy of ICIs remains limited. By downregulating PD-L1 and enhancing CD8⁺ T-cell activity, CTSS inhibition may help overcome the immunosuppressive TME and improve responsiveness to immunotherapy in this challenging subset.

## Conclusion

This study demonstrated that CTSS influences the immunosuppressive microenvironment in CRC by regulating PD-L1 expression through the autophagy pathway. CTSS suppression in CRC cells enhances autophagy-mediated PD-L1 degradation and promotes cytotoxic T-cell activity, underscoring the dual role of CTSS in modulating immune responses. These findings establish CTSS as a promising immunotherapeutic target in CRC. Further clinical studies are warranted to explore the therapeutic potential of CTSS inhibition as a strategy for enhancing the efficacy of immunotherapy in CRC.

## Supplementary Information

Below is the link to the electronic supplementary material.

Supplementary file 1 (PDF 129 KB)

Supplementary file 2 (PDF 529 KB)

Supplementary file 3 (PDF 191 KB)

Supplementary file 4 (PDF 67 KB)

Supplementary file 5 (PDF 67 KB)

Supplementary file 6 (PDF 209 KB)

Supplementary file 7 (PDF 116 KB)

Supplementary file 8 (XLSX 152 KB)

## Data Availability

No datasets were generated or analysed during the current study.

## Abbreviations

AVO: Acidic vesicular organelle
CQ: Chloroquine
CRC: Colorectal cancer
CTLA4: Cytotoxic T-lymphocyte-associated protein 4
CTSS: Cathepsin S
ELISA: Enzyme-linked immunosorbent assay
GEPIA: Gene Expression Profiling Interactive Analysis
GSVA: Gene set variation analysis
ICI: Immune checkpoint inhibitor
IHC: Immunohistochemical
LAG-3: Lymphocyte-activation gene 3
MFI: Median fluorescence intensity
MDC: Monodansylcadaverine
MSI: Microsatellite instability
MHC: Major histocompatibility complex
MSS: Microsatellite stability
PBS: Phosphate-buffered saline
PD-1: Programmed cell death 1
PD-L1: Programmed death-ligand 1
PHA: Phytohemagglutinin
PBMC: Peripheral blood mononuclear cell
shRNA: Short hairpin RNA
siRNA: Small interfering RNA
TCGA: The Cancer Genome Atlas
TME: Tumor microenvironment
TIM-3: T-cell immunoglobulin and mucin-domain containing-3
