# Supplementary material for: Cathepsin S regulates antitumor immunity through autophagic degradation of PD-L1 in colorectal cancer cells
Source: Cancer Immunol Immunother. 2025 Aug 12;74(9):287. doi: 10.1007/s00262-025-04140-x (PMC12343434; doi:10.1007/s00262-025-04140-x)
Supplement: Supplementary file 1 — (PDF 129 KB) [file 262_2025_4140_MOESM1_ESM.pdf]

Supplementary Figure 1

A

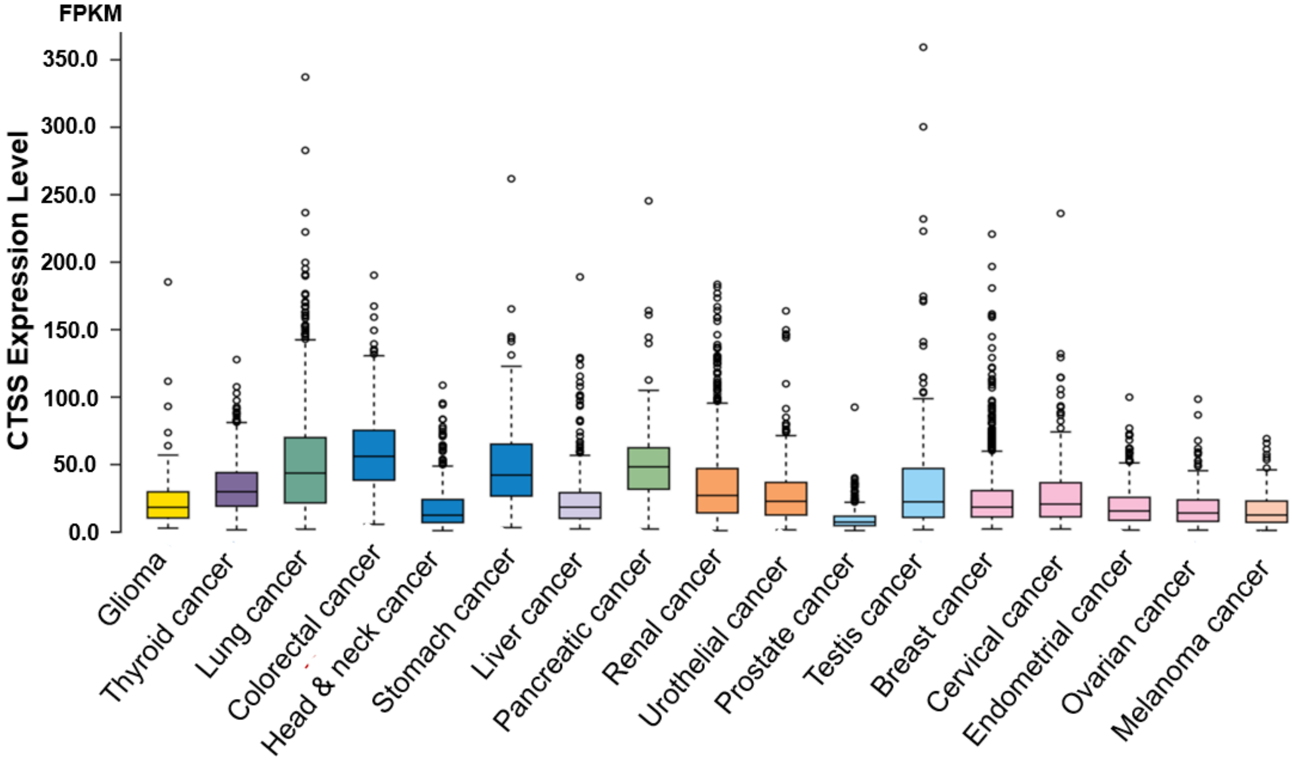

B

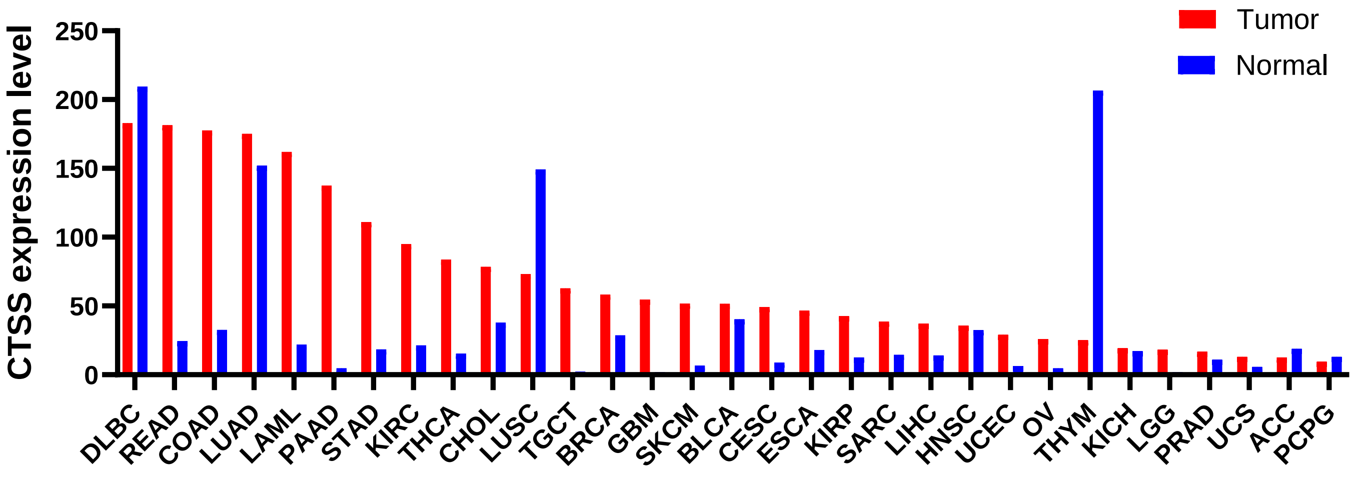

Caption: CTSS upregulation in CRC tissues. (A) TCGA transcriptome data indicated that CTSS expression was highest in CRCs among various types of human cancer. Gene expression boxplots were generated using the Human Protein Atlas platform. (B) CTSS expression was significantly higher in CRC tissues than in normal colon mucosa, as shown in TCGA data. Bar histograms of CTSS expression across different tumor and normal tissues were obtained from the GEPIA platform. Abbreviations: CRC, colorectal cancer; COAD, colon adenocarcinoma; FPKM, fragments per kilobase per million; GEPIA, Gene Expression Profiling Interactive Analysis; READ, rectal adenocarcinoma.
