# Supplementary material for: Cathepsin S regulates antitumor immunity through autophagic degradation of PD-L1 in colorectal cancer cells
Source: Cancer Immunol Immunother. 2025 Aug 12;74(9):287. doi: 10.1007/s00262-025-04140-x (PMC12343434; doi:10.1007/s00262-025-04140-x)
Supplement: Supplementary file 2 — (PDF 529 KB) [file 262_2025_4140_MOESM2_ESM.pdf]

Supplementary Figure 2

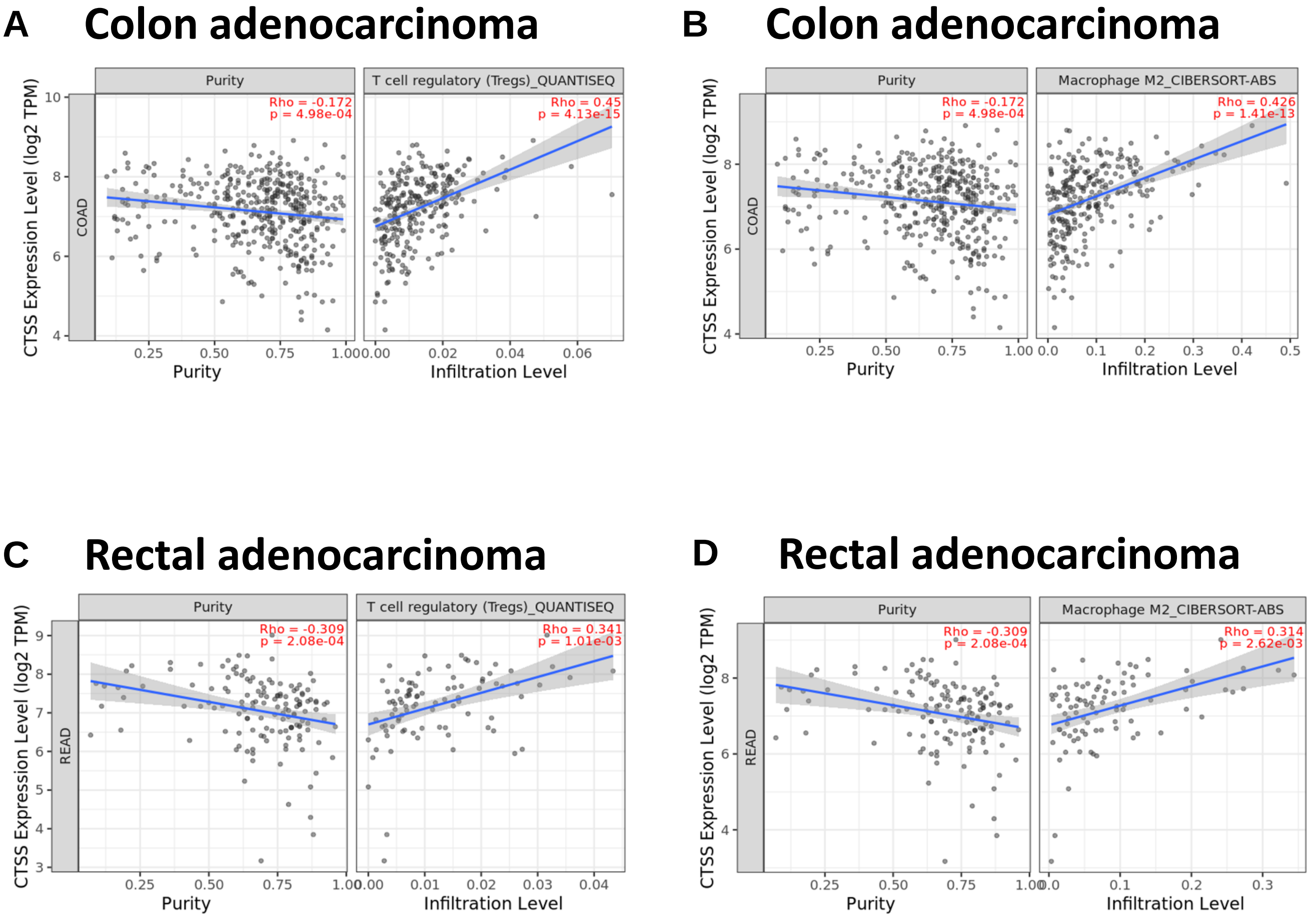

Caption: Positive correlation between CTSS expression and immune cell infiltration in the CRC microenvironment, as analyzed using the TIMER database. (A, B) In COAD, CTSS expression was positively correlated with regulatory T cells and M2 macrophages. (C, D) In READ, similar correlations were observed with regulatory T cells and M2 macrophages. Abbreviations: COAD, colon adenocarcinoma; READ, rectal adenocarcinoma.
