# Supplementary material for: Cathepsin S regulates antitumor immunity through autophagic degradation of PD-L1 in colorectal cancer cells
Source: Cancer Immunol Immunother. 2025 Aug 12;74(9):287. doi: 10.1007/s00262-025-04140-x (PMC12343434; doi:10.1007/s00262-025-04140-x)
Supplement: Supplementary file 3 — (PDF 191 KB) [file 262_2025_4140_MOESM3_ESM.pdf]

Supplementary Figure 3

TCGA colon and rectal adenocarcinoma

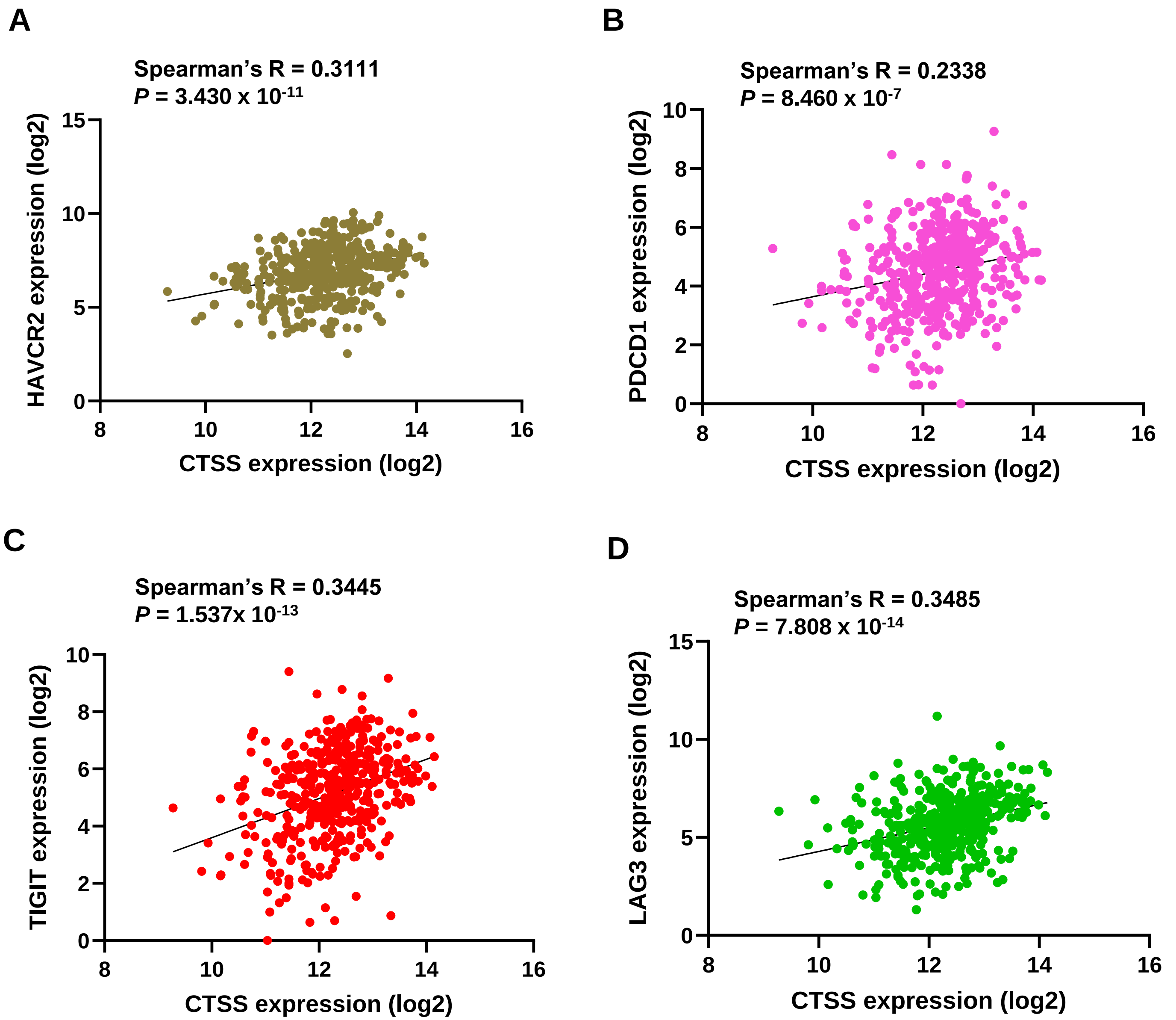

Caption: Correlation between CTSS and immune checkpoint expression in CRC tissues. CTSS expression exhibited positive correlations with multiple immune checkpoint molecules in CRC transcriptomic data from TCGA. (A) HAVCR2, (B) PDCD1, (C) TIGIT and (D) LAG3 expression levels were each positively associated with CTSS expression.
