# Supplementary material for: Cathepsin S regulates antitumor immunity through autophagic degradation of PD-L1 in colorectal cancer cells
Source: Cancer Immunol Immunother. 2025 Aug 12;74(9):287. doi: 10.1007/s00262-025-04140-x (PMC12343434; doi:10.1007/s00262-025-04140-x)
Supplement: Supplementary file 5 — (PDF 67 KB) [file 262_2025_4140_MOESM5_ESM.pdf]

Supplementary Figure 5

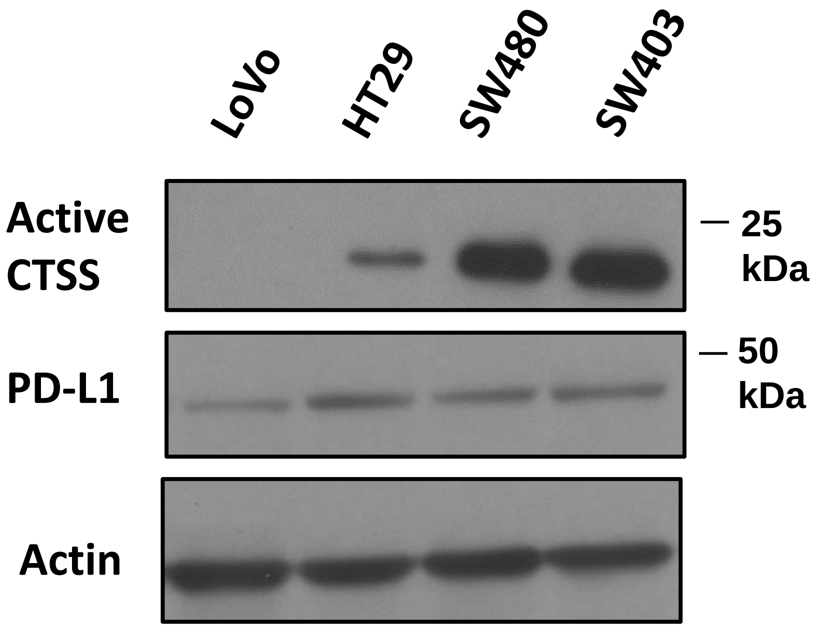

Caption: Differential expression levels of CTSS and PD-L1 in commonly used colorectal cancer cell lines.
