# Supplementary material for: Cathepsin S regulates antitumor immunity through autophagic degradation of PD-L1 in colorectal cancer cells
Source: Cancer Immunol Immunother. 2025 Aug 12;74(9):287. doi: 10.1007/s00262-025-04140-x (PMC12343434; doi:10.1007/s00262-025-04140-x)
Supplement: Supplementary file 6 — (PDF 209 KB) [file 262_2025_4140_MOESM6_ESM.pdf]

Supplementary Figure 6

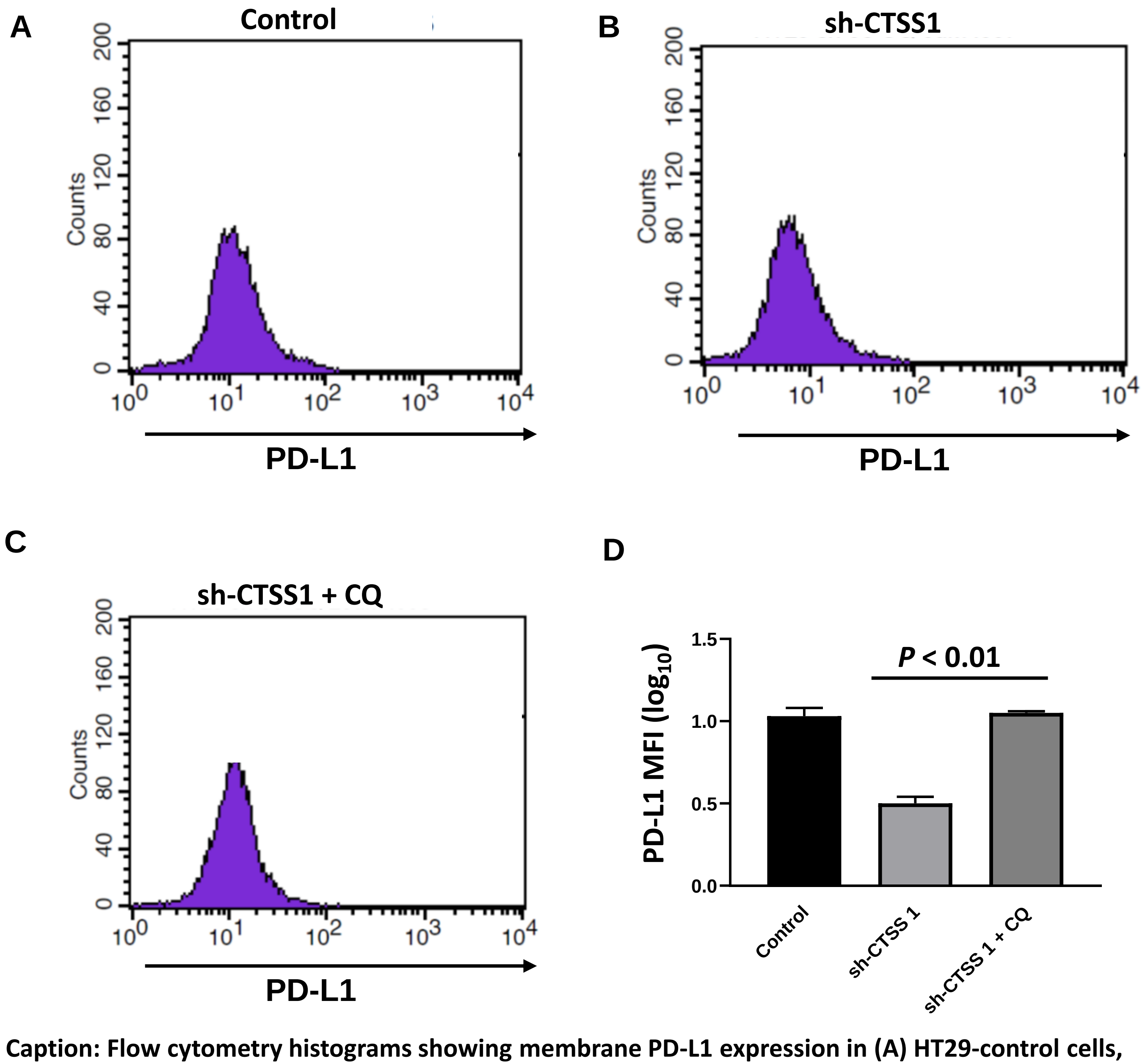

Caption: Flow cytometry histograms showing membrane PD-L1 expression in (A) HT29-control cells, (B) HT29-sh-CTSS cells, and (C) HT29-sh-CTSS cells treated with chloroquine (CQ). (D) Quantification of MFI, presented as mean $\pm$ SD from three independent experiments. Cells were seeded in six-well plates at a density of 2×10^5 cells/well and treated with 5 µM CQ for 48 hours after 24 hours of initial incubation. Abbreviations: CQ, chloroquine; MFI, median fluorescence intensity.
