# Supplementary material for: Cathepsin S regulates antitumor immunity through autophagic degradation of PD-L1 in colorectal cancer cells
Source: Cancer Immunol Immunother. 2025 Aug 12;74(9):287. doi: 10.1007/s00262-025-04140-x (PMC12343434; doi:10.1007/s00262-025-04140-x)
Supplement: Supplementary file 7 — (PDF 116 KB) [file 262_2025_4140_MOESM7_ESM.pdf]

Supplementary Figure 7

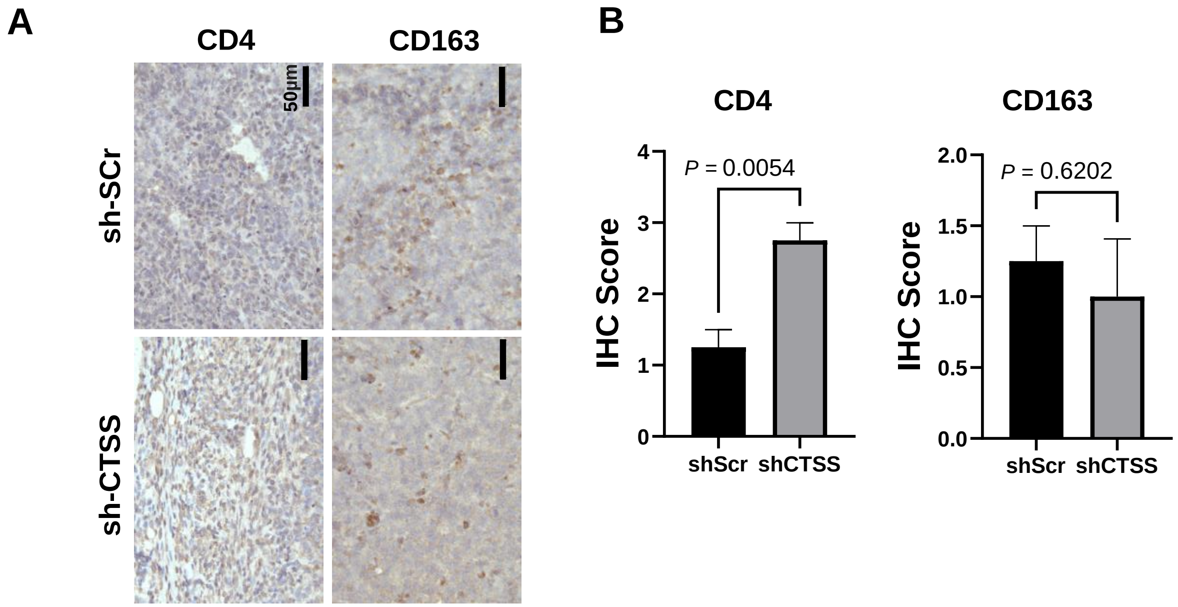

Caption: CTSS-mediated immune modulation in MC38 tumors. (A) Representative immunohistochemical staining of MC38 tumor sections for $CD4^{+}$ T cells and $CD163^{+}$ M2 macrophages. Scale bar: 50 μm. (B) Quantification of CD4 and CD163 immunopositive cells in tumor tissues from sh-Scr and sh-CTSS groups. Data are represented as mean ± SD and were analyzed using a two-tailed Student's t test. P values are indicated.
